# Associating transcriptomics data with inflammatory markers to understand tumour microenvironment in hepatocellular carcinoma

**DOI:** 10.1002/cam4.4941

**Published:** 2022-06-18

**Authors:** Basak Bahcivanci, Roshan Shafiha, Georgios V. Gkoutos, Animesh Acharjee

**Affiliations:** ^1^ College of Medical and Dental Sciences, Institute of Cancer and Genomic Sciences, Centre for Computational Biology University of Birmingham Birmingham UK; ^2^ Institute of Translational Medicine University Hospitals Birmingham NHS Foundation Trust Birmingham UK; ^3^ NIHR Surgical Reconstruction and Microbiology Research Centre University Hospital Birmingham Birmingham UK; ^4^ MRC Health Data Research UK (HDR UK) Birmingham UK; ^5^ NIHR Experimental Cancer Medicine Centre Birmingham UK

**Keywords:** gene signature, hepatocellular carcinoma, immune deconvolution, tumor microenvironment

## Abstract

**Background:**

Liver cancer is the fourth leading cause of cancer‐related death globally which is estimated to reach more than 1 million deaths a year by 2030. Among liver cancer types, hepatocellular carcinoma (HCC) accounts for approximately 90% of the cases and is known to have a tumour promoting inflammation regardless of its underlying aetiology. However, current promising treatment approaches, such as immunotherapy, are partially effective for most of the patients due to the immunosuppressive nature of the tumour microenvironment (TME). Therefore, there is an urgent need to fully understand TME in HCC and discover new immune markers to eliminate resistance to immunotherapy.

**Methods:**

We analyse three microarray datasets, using unsupervised and supervised methods, in an effort to discover signature genes. First, univariate, and multivariate, feature selection methods, such as the Boruta algorithm, are applied. Subsequently, an optimisation procedure, which utilises random forest algorithm with three dataset pairs combinations, is performed. The resulting optimal gene sets are then combined and further subjected to network analysis and pathway enrichment analysis so as to obtain information related to their biological relevance. The microarray datasets were analysed via the MCP‐counter, CIBERSORT, TIMER, EPIC, and quanTIseq deconvolution methods and an estimation of cell type abundances for each dataset sample were identified. The differences in the cell type abundances, between the adjacent and tumour sample groups, were then assessed using a Wilcoxon Rank Sum test (*p*‐value < 0.05).

**Results:**

The optimal gene signature sets, derived from each of the data pairs combination, achieved AUC values ranging from 0.959 to 0.988 in external validation sets using Random Forest model. CLEC1B and PTTG1 genes are retrieved across each optimal set. Among the signature genes, PTTG1, AURKA, and UBE2C genes are found to be involved in the regulation of mitotic sister chromatid separation and anaphase‐promoting complex (APC) dependent catabolic process (adjusted *p*‐value < 0.001). Additionally, the application of deconvolution algorithms revealed significant changes in cell type abundances of Regulatory T (Treg) cells, M0 and M1 macrophages, and T CD8^+^ cells between adjacent and tumour samples.

**Conclusion:**

We identified ECM1 gene as a potential immune‐related marker acting through immune cell migration and macrophage polarisation. Our results indicate that macrophages, such as M0 macrophage and M1 macrophage cells, undergo significant changes in HCC TME. Moreover, our immune deconvolution approach revealed significant infiltration of Treg cells and M0 macrophages, and a significant decrease in T CD8^+^ cells and M1 macrophages in tumour samples.

## Graphical abstract

Workflow for transcriptomics analysis. We associated transcriptomics data with inflammatory markers to understand the tumour microenvironment in hepatocellular carcinoma using a machine learning algorithm. We identified genes involved in the coagulation cascades pathways and their impact on regulatory T cells.

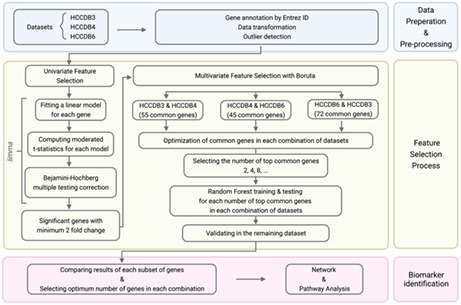

## 1 BACKGROUND

Liver cancer is the fourth leading cause of cancer‐related death globally which is estimated to reach more than 1 million deaths in 2030.^1^ The most common type of liver cancer is hepatocellular carcinoma (HCC), which accounts for approximately 90% of the cases.^2^ Chronic viral infection with hepatitis B (HBV) and hepatitis C (HCV) are the main causes for HCC development. On the other hand, other non‐viral underlying aetiologies, such as non‐alcoholic steatohepatitis (NASH) and non‐alcoholic fatty liver disease (NAFLD)^3^ driven by obesity and metabolic syndrome, are rapidly becoming a common risk factor of HCC in the western countries.^1^, ^4^ Recent findings show that NASH has become the second cause of HCC in the United States which accounts for 18% of HCC cases with an 8‐fold increase since 2002.^4^ Curative treatments, such as surgical resection and radiofrequency ablation (RFA) in early stages of HCC, are heavily affected by recurrence rates and transplantation is governed by extremely thorough oncological criteria.^5^ Moreover, HCC diagnosis is often significantly delayed.^6^ Therefore, more than 70% of the patients are diagnosed as unresectable and the effectiveness of the current therapies are limited for advanced stage disease.^6^, ^7^

However, in recent years, it has been hypothesised that reprogramming the tumour microenvironment (TME) using immunotherapeutic drugs, due to its immune‐rich contexture, could shape the future of HCC treatment.^5^, ^8^ Even though the underlying aetiology changes, tumour promoting inflammation remains a common characteristic for the development of HCC.^5^ Therefore, in the past several years, HCC has garnered more attention for the optimisation of treatment combinations with immune‐oncology.^8^

Nonetheless, understanding the complex mechanism of TME remains a challenge, and current immunotherapies have shown partial success due to its immunosuppressive nature.^5^, ^7^ Currently, most studies are conducted by immunohistochemistry‐based analysis with a focus of one or two cell types.^9^ However, TME is characterised by complex interactions between several cell types.^9^ Thus, there is an urgent need to fully discover the functional characteristics of TME to eliminate resistance to immunotherapy and improve the efficacy of the drugs.^5^, ^8^, ^9^

In this study, we applied immune deconvolution methods and identified associated transcriptomics signatures, derived from the analysis of three microarray datasets, with their immune markers. To generate transcriptomics signatures, we conducted feature selection with univariate analysis and filtered differentially expressed genes between adjacent and normal tissues. We further applied multivariate feature selection to each dataset via Boruta algorithm. Then, the optimal gene set was obtained following an optimisation procedure, which utilised a Random Forest (RF) classifier. On the other hand, gene expression profiles of three microarray datasets were deconvoluted by the MCP‐counter, CIBERSORT, TIMER, EPIC, and quanTIseq immune deconvolution methods to estimate cell contents in the samples. Potential associations between identified transcriptomics signatures and immune markers were then obtained via a pathway enrichment analysis.

## 2 METHODS

### 2.1 Data collection and pre‐processing

Three microarray datasets were downloaded from the Database of Hepatocellular Carcinoma Expression Atlas (HCCDB),^10^ on March 2021, based on the balance as well as the amount of sample sizes they contained (Table 1). HCCDB extracts probe values (log2 intensity) and probe annotations of datasets from raw files of Gene Expression Omnibus (GEO) database. In this study, gene symbols are re‐annotated by using their Entrez IDs via *AnnotationDbi* package in R.^11^ Moreover, genes with a median absolute deviation less than 0.5 are removed. Only HCCDB4 has one missing value, which was imputed with its group mean. Lastly, datasets are auto‐scaled prior to analysis. Table 1 summarises the information for the datasets which are used in the analysis.

**TABLE 1:** HCCDB datasets

Dataset ID	Source	Platform	Number of adjacent samples	Number of HCC samples
HCCDB3	GSE25097	Rosetta/Merck Human RSTA Affymetrix 1.0 microarray	243	268
HCCDB4	GSE36376	Illumina HumanHT‐12 v4.0 expression beadchip	193	240
HCCDB6	GSE14520 (GLP3721)	Affymetrix Human Genome U133A 2.0 Array	220	225

### 2.2 Principal component analysis (PCA) and t‐distributed stochastic neighbour embedding (t‐SNE)

Principal component analysis (PCA) is a widely used dimension reduction approach. It constructs a new set of uncorrelated variables called principal components (PCs) which contain the linear combinations of the original variables (i.e., gene expressions). PCs can summarise the data with less dimensions which enables the visualisation of high dimensional datasets.

In our analysis, log‐transformed and auto‐scaled datasets were used to identify potential outliers via PCA with confidence ellipsoids. These ellipsoids, for each group, were drawn by calculating the Mahalanobis distances of PCs, which were approximately Chi‐square distributed. The Mahalanobis distance was calculated by using the inverse of the variance–covariance matrix of the data, using PCs rather than original variables. By using these distances and the centre point of PCs, a 95% confidence ellipse was drawn for the chi‐squared distributed Mahalanobis PCs distance.

On the other hand, t‐distributed stochastic neighbour embedding (t‐SNE) is an embedding technique which preserves local similarities and is an unsupervised non‐linear dimensionality reduction and data visualisation technique. Unlike PCA, it caters hyperparameter tuning, for example, perplexity.

### 2.3 Univariate feature selection by *limma*

**FIGURE 1:**
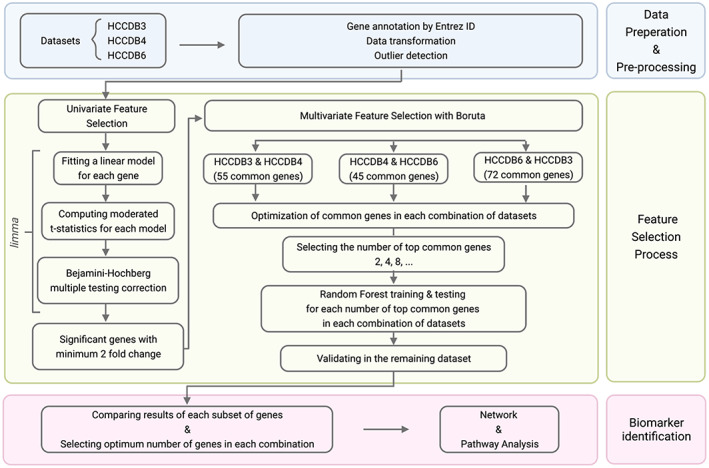
Workflow for transcriptomics analysis.

The *limma* package^12^ was used to obtain the differentially expressed genes between adjacent and tumour samples. After fitting a linear model for each gene, an empirical Bayes method was used to compute moderated t‐statistics estimating the overall variability over all genes and adjusting for high and low variability genes. After obtaining the significant genes with adjusted *p*‐value < 0.05, the remaining genes were filtered with a 2‐fold change cut‐off between two groups.^13^, ^14^ Figure 1 presents schematically the *limma* based workflow.

### 2.4 Random forest

Random forests (RFs) are an ensemble of decision trees in which classification is performed by voting of many weak unbiased classifiers.^15^, ^16^, ^17^, ^18^ RFs cater for the capture of complex interactions as well as for low bias due to the lack of pruning, rendering them well suited for microarray analysis.^17^, ^19^

RFs typically require a tuning of the number of predictors to select in each split (mtree) and of the number of trees to grow (*ntree*) hyperparameters. In our study, *caret,* ^20^ a R package, was used for RF modelling. A grid search method was used for the hyperparameter selection and subsequently a 10‐fold cross‐validation with 10 repeats, for each training set, was applied.

### 2.5 Boruta algorithm for feature selection

Boruta is a wrapper method built around RFs for feature selection.^18^ A “shadow attribute” is created for each attribute obtained by shuffling the values of the original ones across samples.^18^ Then a classification task over the extended system, using both original and shadow attributes, is performed and the importance of all attributes is computed. The maximum Z‐score, among the shadow attributes (MZSA) is used as a reference when deciding the significance of the original attribute's importance.^18^ Depending on attribute significant importance in relation to Boruta considers the attributes, which have significantly higher importance than MZSA, attributes are categorised as “important”, and “unimportant”.

In the analysis, each dataset, including differentially expressed genes, was partitioned into testing (%70) and training (%30) sets, and the input seed for *set. seed* function was retained so as to reproduce the same partitions for each data sets throughout the analysis. Only the features which were assigned as “important” were kept for each dataset, and “unimportant” features were discarded.

The square root of features was used for the *mtry* hyperparameter, and 1500 trees were used.

### 2.6 Optimization of selected genes with random forest

**FIGURE 2:**
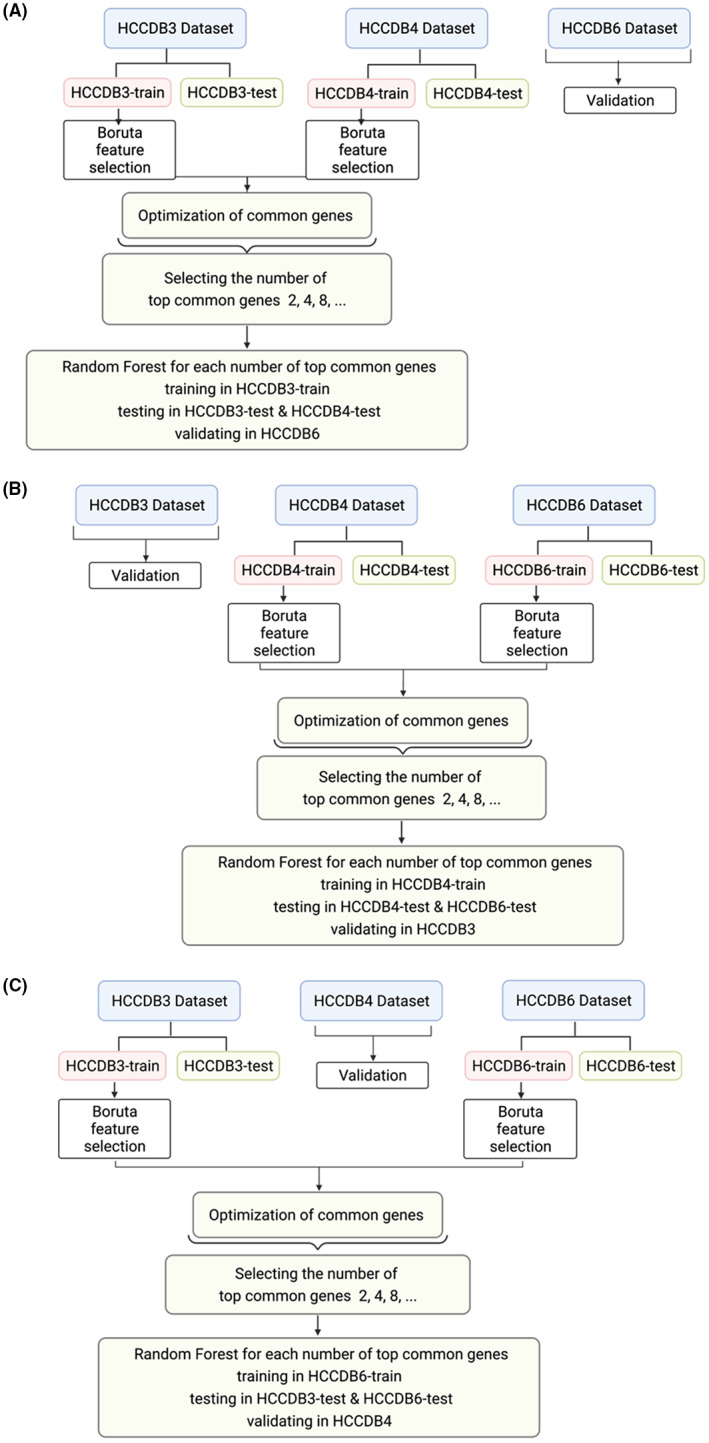
Common gene optimisation process in each pair of data sets. (A) Combination 1. (B) Combination 2. (C) Combination 3.

Common genes between data set pairs, confirmed as important by Boruta, were then obtained. To reduce the bias of the selection criteria, a number of data set pairs combinations were considered (Figure 2) to ensure that each dataset was used only once as a train set and validation set.

The optimisation procedure was performed by selecting the top common genes in each combination of data set pairs in a geometric progression manner (i.e., 2, 4, 8, 16, ...). For each combination, a RF classifier was trained, tested, and validated (Figure 2). Then, the optimal set of genes was selected based on AUC value differences for each dataset combination. The resulting three optimal gene sets were then combined (optimal gene set).

### 2.7 Network analysis

After obtaining the optimal set of genes, *qgraph* package^21^ is used to construct the partial correlation network of the optimal set. Package computes the correlation of two variables after removing the other variables' effect from the system. By this way, we eliminated misleading associations which might arise from confounding variables. Furthermore, Benjamini‐Hochberg adjusted *p*‐values were used to decrease false discovery rate for the edges in the network with *q*‐value <0.05.

### 2.8 Pathway enrichment analysis

The derived optimal gene set was then subjected to GO Biological Process 2021 (GO: BP) and KEGG Human 2021 pathway enrichment analysis using *enrichR* package.^22^, ^23^

### 2.9 Immune deconvolution algorithms

The *Immunedeconv* R package^24^ was used to deconvolute samples (Additional file 1: Table S1). For each method, a two‐sided Wilcoxon rank‐sum test (also called Mann–Whitney *U* test) was performed to compare abundances between adjacent and tumour (HCC) samples for each estimated cell type with a significance level 0.05 so as to analyse alteration in immune cell type content between adjacent and tumour tissues.

### 2.10 Microenvironment cell populations counter (MCP‐counter)

Microenvironment cell populations counter (MCP‐counter) is a marker‐based method which quantifies the absolute abundance of eight immune cells and two stromal cell populations.^14^

This method allows for inter‐sample comparisons but since the computed abundance scores are expressed in arbitrary units and not fractions no comparisons between cell types are possible. For our analysis, the parameters of the functions used within the *immunedeconv* package were set to default.

## 3 CIBERSORT

CIBERSORT is a deconvolution‐based method to characterise cell compositions of tissue samples by using their gene expression profiles.^25^

Cell‐specific gene expression signatures are stored in a matrix called LM22.^25^ LM22 is used as a reference gene expression matrix considered as a minimum representation for each cell population. Based on these values, the method infers the unknown cell fractions for each cell type in a given mixture using nu‐support vector regression (ν‐SVR).^25^, ^26^

CIBERSORT requires expression data to be in non‐log linear space. Therefore, for our analysis, data was transformed into the recommended format.^27^ Following the submission of the corrected and the selection of the LM22 matrix as a signature matrix, the quantile normalisation was disabled since the datasets were already normalized. The number of permutations for the statistical analysis were set to 500 as to estimate robust *p*‐values.

## 4 TIMER

TIMER estimates the abundance of six tumour infiltrating immune cell populations for 23 cancer types including liver hepatocellular carcinoma (LIHC).^28^ TIMER infers the abundance of the six immune cell types by fitting a constrained least squares regression for each sample. It only allows for inter sample comparisons (for same cancer type samples) since calculated abundance scores are in arbitrary units.

During analysis using the *immunedeconv* R package, the parameter indication is set as “LIHC” for each sample to indicate that samples are from hepatocellular carcinoma. Microarray datasets were used without log‐transformation.

### 4.1 Estimating the proportion of immune and cancer cells (EPIC)

EPIC is a method that estimates the fraction of immune and cancer cell populations from bulk tumour gene expression data.^29^ Unlike other methods, EPIC is based on two reference gene expression profiles which are single‐cell or bulk RNA‐seq data.^29^ The first one is built from single‐cell RNA‐seq data of melanoma patients, and the other is derived from peripheral blood samples of sorted six immune cells.

Although uncharacterised cell types are not accounted for in the signature matrix, EPIC generates absolute scores which is provided not only for characterised cells but also for “other” cell types (i.e., cancer cells) with unknown signatures. Therefore, these scores can refer to the total cells in a sample and can be used as absolute fractions.^29^, ^30^ This allows EPIC scores to be used for both within and between sample comparisons for cell types.

Analysis was performed using the *immunedeconv* package. The *scale_mrna* parameter of the deconvolute function was set to TRUE, estimating and correcting for the different cell type mRNA content.^24^ In our case, EPIC algorithm was not able to predict these values for CAFs and Endothelial cells. Therefore, the calculated absolute scores were not considered as absolute cell proportions in our analysis to avoid biased interpretations about fractions.

### 4.2 The quantification of the tumour immune contexture from human RNA‐seq data (quanTIseq)

quanTIseq is a deconvolution method for bulk RNA‐seq data.^31^ The *immunedeconv* package can run within quanTIseq using a mode which is optimised for microarray data.^24^

quanTIseq infers the cell type fractions via constrained least squares regression,^31^ in a similar way to other deconvolution‐based methods. Similar to EPIC, it computes absolute scores which can be used as cell fractions. This allows quanTIseq to be used for both within and between sample comparisons with the condition of being able to predict the proportions of mRNA per cell value.

## 5 RESULTS

### 5.1 Transcriptomics signature identification

#### 5.1.1 Univariate feature selection analysis

**FIGURE 3:**
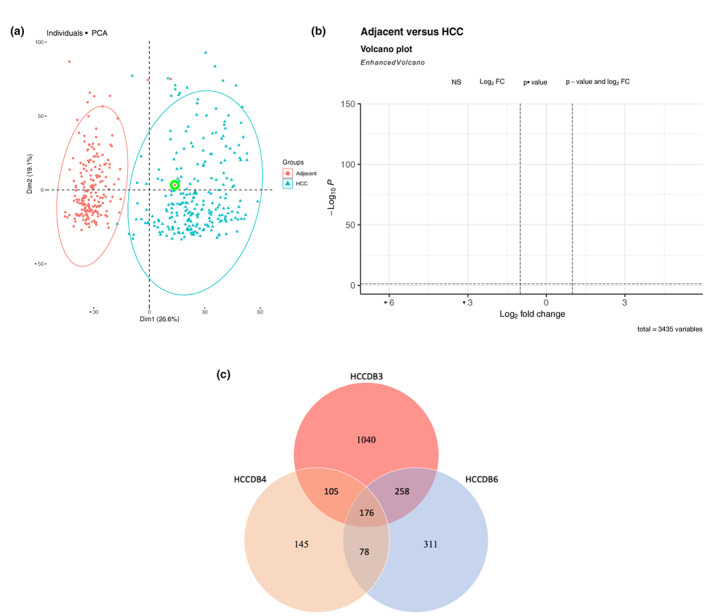
PCA plot and the plots related to differentially expressed genes. (A) PCA plot of HCCDB4 dataset with adjacent Sample 121 in the green circle. (B) Volcano plot of HCCDB4 dataset. (C) Venn diagram of differentially expressed genes.

Prior to univariate feature selection, an outlier detection method, based on PCA with Mahalanobis distances and t‐SNE, was applied to explore data and potential outliers. Figure 3A illustrates the HCCDB4 data PCA plot with one potentially misclassified sample (adjacent Sample 121) within the HCC cluster. Similarly, the t‐SNE technique also identified adjacent Sample 121 within the HCC group reporting it significantly far from other HCCDB4 sample groups (Additional file 2: Figure S1a). In addition, we also identified one potentially misclassified sample (adjacent Sample 283) within the HCC cluster in HCCDB3 data via t‐SNE and PCA analysis (Additional file 2: Figure S1b,c). Additional file 2: Figure S1d displays the PCA plot showing one sample (adjacent Sample 88) at the bottom far left of the figure in HCCDB6 data. On the other hand, t‐SNE plot highlighted many samples distributed within both groups (Additional file 2: Figure S2a). Therefore, by evaluating both the PCA and t‐SNE results, we identified and removed the adjacent Sample 88 of HCCDB6 data as a potential outlier. As a result, these mentioned samples were removed from our downstream analysis. After removing potential outliers, a univariate analysis was performed using the *limma* package.^12^ As a result, differentially expressed genes were obtained, and they were further filtered with a 2‐fold change cut‐off between two groups. A volcano plot is presented in Figure 3B and Additional file 2: Figure S2b, c. In these volcano plots, gray dots represent the genes that were found to be statistically insignificant with an adjusted *p*‐value > 0.05. Blue dots denote the genes which were found to be statistically significant, and red ones highlight genes that were found to be both significant and have a fold change greater than 2. Moreover, Figure 3c shows the Venn diagram of the resulting differentially expressed gene (DEGs) set of each dataset.

#### 5.1.2 Feature selection with Boruta algorithm and optimisation of selected genes

Out of the 1579 DEGs, 386 genes were confirmed as important for HCCDB3‐train set by Boruta. On the other hand, 203 out of 504 DEGs were found as important for HCCDB4‐train set. Lastly, for HCCDB6‐train set, 177out of 823 DEGs were confirmed as important.

In total, 13 genes were obtained by the optimisation process. Moreover, the analysis showed CLEC1B and PTTG1 genes as commonly selected genes in all optimized data pairs with significantly decreased and increased expression levels, respectively, for all datasets (Additional file 2: Figure S3†S8).

**FIGURE 4:**
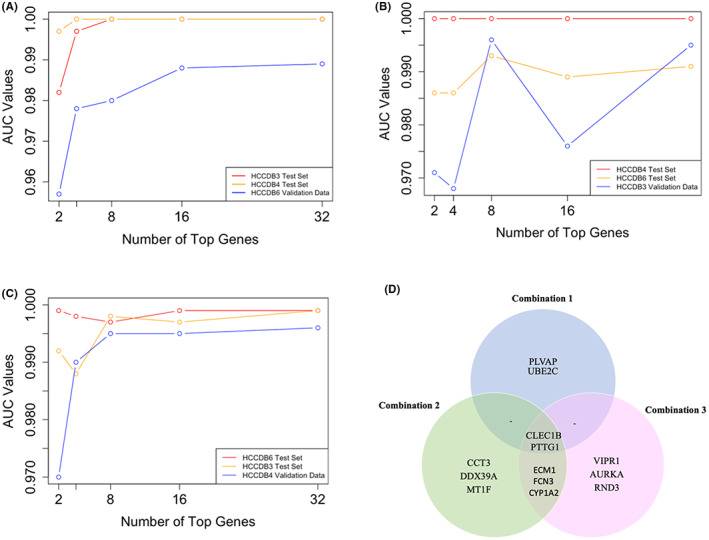
AUC values of random forests. (A) AUC values for the top common genes obtained from HCCDB3 and HCCDB4 dataset pairs (Combination 1). (B) AUC values for the top common genes obtained from HCCDB4 and HCCDB6 dataset pairs (Combination 2). (C) AUC values for the top common genes obtained from HCCDB3 and HCCDB6 dataset pairs (Combination 3). (D) Venn diagram of top common gene sets selected via random forest AUC values.

Figure 4A, B, c show the AUC values of top common gene sets for each combination. The top 4 common genes for Combination 1, and top 8 common genes for Combination 2 and Combination 3 were selected based on the most significant AUC values increase. Table 2 shows the number of common genes before and after the optimisation process and Figure 4d presents a Venn diagram of the optimised gene sets.

**TABLE 2:** Number of common genes before and after optimisation process

Combination of data set pairs^a^	Number of common genes between data set pairs	Number of common genes after optimisation process
HCCDB3 and HCCDB4	55	4
HCCDB4 and HCCDB6	45	8
HCCDB3 and HCCDB6	72	8

^**a**^ Datasets with features after feature selection process.

Down and up‐regulation information for the optimal genes for each dataset are provided in the Additional file 2: Figure S3‐S8.

#### 5.1.3 Network and pathway enrichment analysis

**FIGURE 5:**
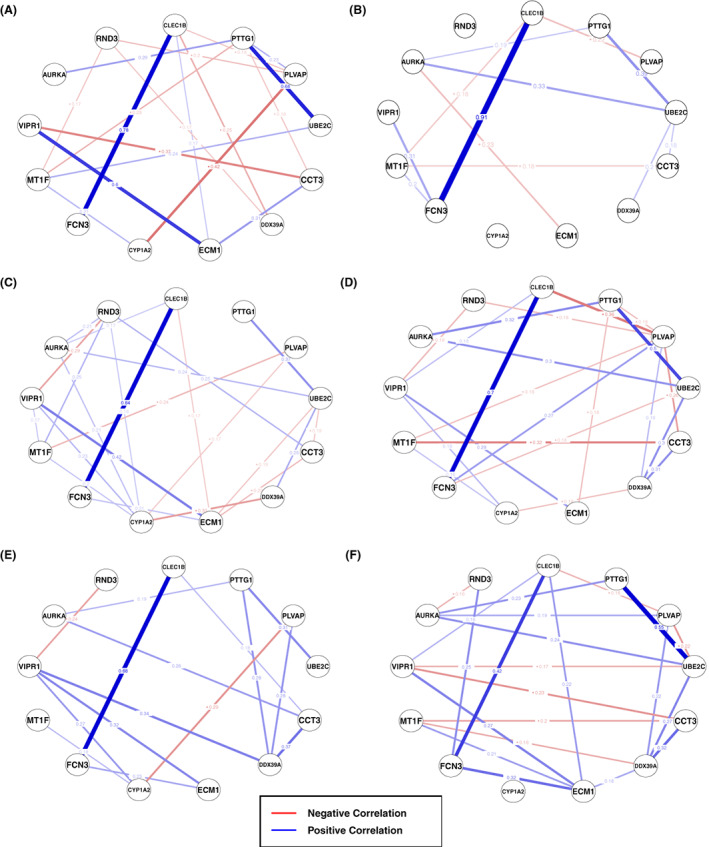
Partial network analysis. (A) Partial correlation plot of adjacent samples of HCCDB3 dataset. (B) Partial correlation plot of HCC samples of HCCDB3 dataset. (C) Partial correlation plot of adjacent samples of HCCDB4 dataset. (D) Partial correlation plot of HCC samples of HCCDB4 dataset. (E) Partial correlation plot of adjacent samples of HCCDB6 dataset. (F) Partial correlation plot of HCC samples of HCCDB6 dataset. The width of the lines relates to the extent of the correlation.

A partial correlation network was used to analyse the change in network structure between adjacent and HCC samples (Figure 5) revealing the degree of genes behaving differently between the adjacent and HCC groups. The absolute degree difference for each gene between adjacent and HCC groups were calculated. Then, the genes were ranked, based on the number of connectivity (degree), in decreasing order and only the upper quartile genes were further considered (Additional file 3: Table S1‐S3). Network analysis showed that the degree of PTTG1, PLVAP, ECM1, UBE2C, and FCN3 genes were increased in HCCDB3 dataset (Figure 5A, B). On the other hand, Figure 5c, d illustrates that the degree of CYP1A2, ECM1, and RND3 were increased in the HCCDB4 dataset, and contrary to HCCDB3 dataset, the degree of PLVAP and PTTG1 were decreased in the HCCDB4 dataset. Lastly, in HCCDB6 dataset, only the degree of CYP1A2 gene were increased (Figure 5.e, f). However, the degrees of UBE2C, ECM1, and CLEC1B genes were decreased.

**FIGURE 6:**
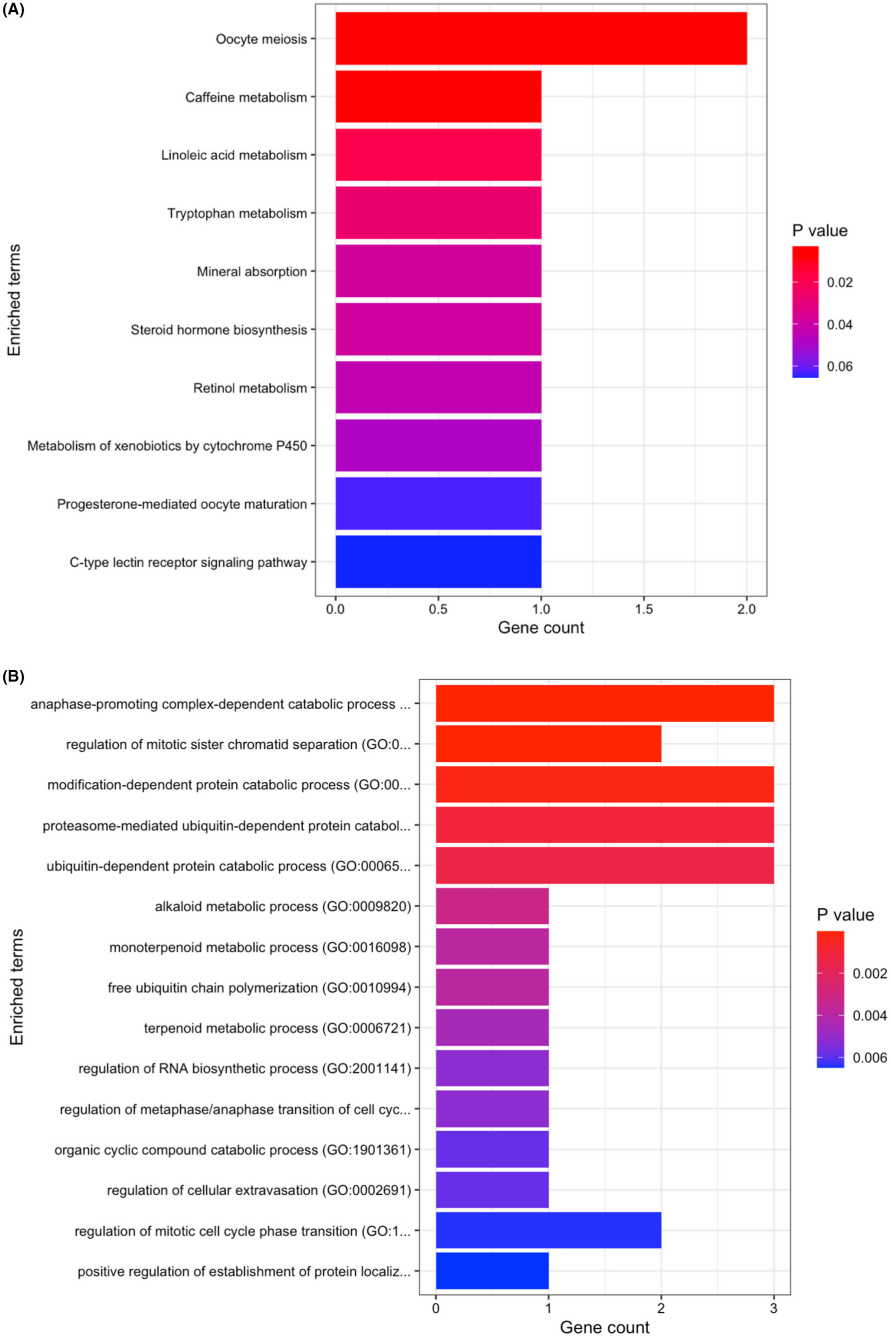
Enrichment analysis plots. a KEGG pathway enrichment plot of selected 13 marker genes. b GO: BP results of selected 13 marker genes.

Figure 6A illustrates the KEGG Human 2021 pathways in which the 13 signature genes are involved. The PTTG1 and AURKA genes were primarily involved in oocyte meiosis related pathways. Moreover, the CYP1A2 gene was found to be significantly involved in caffeine metabolism pathway via KEGG pathway analysis (version 2021). In addition to KEGG pathway analysis, GO Biological Process (GO: BP, version 2021) based results revealed the PTTG1, UBE2C, and AURKA genes to be involved in the regulation of mitotic sister chromatid separation and the pathways and the chemical reactions causing protein or peptide bonds breakdown with the highest significance (Figure 6B). We also note that these genes were significantly upregulated across all datasets (Additional file 2: Figure S4, S6, S8). The KEGG Human 2021 pathway analysis table is presented in the Additional file 4: Table S1 and the GO: BP analysis table is provided within the Additional file 4: Table S2.

#### 5.1.4 Microarray based estimation of TME

Deconvolution of the three datasets was achieved using the MCP‐counter, CIBERSORT, TIMER, EPIC, and quanTIseq algorithms. The tumour samples showed significant infiltration by Regulatory T (Treg) cells and naïve Macrophage (M0 macrophage) cells. In addition, decrease in T CD8^+^ cells and Macrophage 1 (M1 macrophage) cells in tumour samples was observed.

Our results showed that there was disagreement between the estimated cell type abundances among datasets and/or methods. However, the M0 macrophage, Treg and uncharacterized cell changes between adjacent and tumour groups were consistent for all datasets and across all available methods.

**FIGURE 7:**
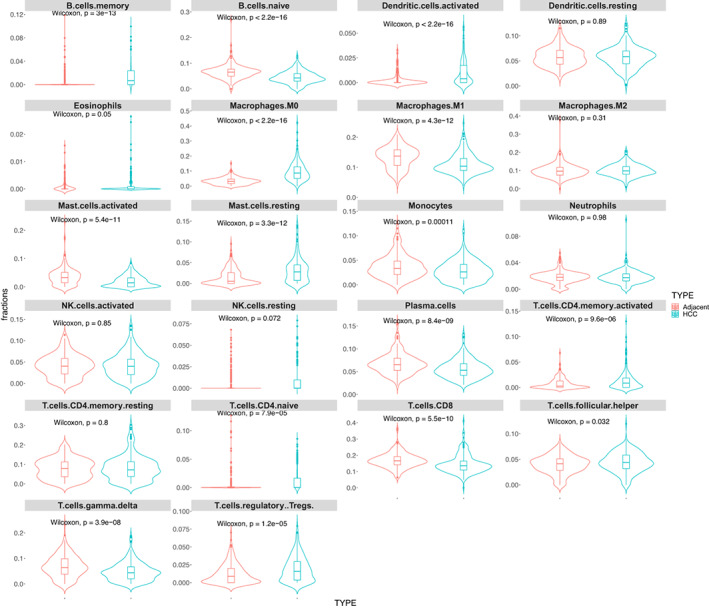
CIBERSORT cell type abundance comparison between adjacent and tumour groups in HCCDB6 dataset. Violin box plots of CIBERSORT show abundance scores for adjacent and tumour (HCC) groups in HCCDB6 dataset. Two‐sided Wilcoxon rank‐sum tests were performed to compare two groups for each cell type with 0.05 significance level. 431 significantly deconvoluted samples out of 444 samples are considered when comparing groups.

CIBERSORT (Figure 7, Additional file 2: Figure S9 and S10) and quanTIseq (Additional file 2: Figure S11–S13) are the only methods that can estimate the M1 macrophages and Treg cells. Both these methods showed a significant decrease in M1 macrophages in tumour samples of all datasets except for the HCCDB3 dataset, which showed no significant change in its CIBERSORT estimation (Additional file 2: Figure S9). However, CIBERSORT deconvoluted only 48 samples significantly out of 511 samples for HCCDB3 data sets, contrary to other datasets. In contrast, the Treg cell type abundance was significantly increased in tumour samples. Similarly, CIBERSORT estimated significant M0 macrophage infiltration in tumour samples for all datasets.

T CD8+ cell abundance, which can be estimated by MCP‐counter (Additional file 2: Figure S14–S16), CIBERSORT, EPIC (Additional file 2: Figure S17–S19) and quanTIseq algorithms, also significantly decreased in tumour samples, except for the HCCDB4 sample in MCP‐counter and CIBERSORT estimations, which showed no significant changes.

**FIGURE 8:**
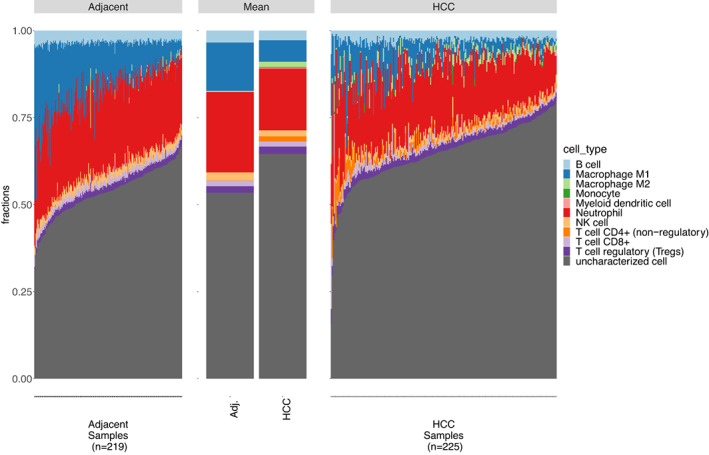
quanTIseq cell type fraction comparisons between adjacent and tumour groups in HCCDB6 dataset. Stacked bar charts of cell type fractions of adjacent and HCC groups are sorted in ascending order of uncharacterized cells (i.e., tumour and stromal) and aggregated by the mean proportion of cell types in each group.

quanTIseq was the only method able to estimate cell fractions, which allowed for within and between sample comparisons. A visualisation of how TME is changing between adjacent and tumoru environments was obtained by plotting stacked bar charts of cell fractions shown in Figure 8 and Additional file 2: Figure S20 and S21. Statistical test results for the significance of changes between groups are given in Additional file 2: Figure S11–S13.

TIMER, on the other hand, requires the specification of cancer type to deconvolute tumor samples. Hence, TIMER was only used to infer TME of tumour samples and the resulting estimates were analysed by considering tumour stages. HCCDB4 was the only dataset that included the tumour stages, which are 1, 2, 3a, 3b, and 3c. For this case, Stage 3a,3b and 3c were merged since their group sample size was very small compared to Stages 1 and 2. The Kruskal Wallis test was then applied to compare the medians of each cell type abundancies between tumour subtypes with 0.05 significance level and allowed for the evaluation of the immune landscape changes between tumour subtypes. There were no significant differences between tumour subtype medians for all cell types and for all datasets (Additional file 2: Figure S22).

Finally, other cell types, for example B cell, cancer associated fibroblast (CAF), Neutrophils, Endothelial, Macrophage 2 (M2 macrophage) etc, showed inconsistent changes between sample groups among datasets and the available methods.

## 6 DISCUSSION

This study employed a univariate analysis and multivariate feature selection framework to identify immune‐related gene signatures associated with HCC. Furthermore, the HCC tumour microenvironment was analysed with five immune deconvolution algorithms to identify potential immune markers.

Following univariate feature selection via the *limma* package, a Boruta feature selection algorithm was used. We used this method since in the similar benchmark analysis, it is demonstrated to be the most stable algorithm compared to the other comparable methods for example: Permutation‐based feature selection, (with and without correction) method, the backward elimination‐based feature selection method in simulated^31^, ^32^ and experimental omics datasets.^32^, ^33^After the feature selection procedure, the optimum set of 13 signature genes was selected via our gene subset optimisation framework and investigated with network analysis and pathway enrichment analysis.

As a result, the significantly upregulated PTTG1 and AURKA genes were found to be involved in oocyte meiosis‐related pathways. Moreover, the CYP1A2 gene was found to be significantly involved in caffeine metabolism pathway via KEGG Human 2021 pathway analysis. Moreover, based on GO: BP analysis, PTTG1, AURKA, and UBE2C genes are found to be involved in the regulation of mitotic sister chromatid separation and anaphase‐promoting complex (APC) dependent catabolic process (adjusted *p*‐ value <0.001). It has previously been reported that, being a specific ubiquitin ligase, APC dependent protein or peptide bonds breakdown has an essential role in cell cycle regulation.^34^ Moreover, its deregulation can lead to genomic instability during tumour progression via genetic mutations which make cancer cells therapeutically impossible to target for later stages.^34^ Recently, Li et al. demonstrated that in hepatitis B Virus (HBV) infection associated HCC, HBV‐induced miR‐122 inhibition leads to upregulated PTTG1 which promotes HCC cell growth and invasion.^35^

In addition to these, we have identified ECM1 gene which is important for wound healing, tissue regeneration, and in disease progressions such as cancer and liver fibrosis.^36^, ^37^ More importantly, ECM1 gene is highlighted to affect immune cell behavior through the interaction of extracellular matrix (ECM) proteins and cell surface receptors which activate cell migration,^36^ proliferation,^37^ and signalling pathways.^38^, ^39^ Moreover, ECM1 gene is recently found to have a crucial function in M1 macrophage polarisation in inflammatory bowel disease after lipopolysaccharide (LPS) treatment.^40^ In this study, Zhang et el. provided experimental evidence on the function of this gene and identified ECM1 as a macrophage‐derived protein in DSS‐induced mouse colitis.^40^

After identification of the gene signature for HCC, we have used five deconvolution algorithms to find immune markers of TME. Our findings showed significant infiltration of Treg cells and M0 macrophage cells in tumour samples. Moreover, a significant decrease in T CD8^+^ cells and M1 macrophages in tumour samples was detected. These findings were consistent among all of the datasets examined and across all of the deconvolution algorithms assessed. The differences of immune infiltration of immune cell types between disease stages of 1, 2, 3a, 3b and 3c were analysed with TIMER.

Specifically, during our HCC analysis, stage 3a, 3b and 3c were merged as Stage 3 to overcome the limitation of uneven class distribution of stages. The differences of immune cell type abundances between disease stages were not significant according to HCCDB4 dataset. Nevertheless, the generalisation of the results requires more data which include stage information of the patients to understand the effect of the disease stage in TME.

Similar to our study, a feature selection procedure followed by an immune marker identification was also performed in other multiple cancer types like Colon and Bladder cancer types.^41^ In this study, a random forest‐based feature selection and deconvolution analysis with the TIMER algorithm were performed in order to find immune‐related markers.^41^

Herein, following the gene signature and the immune marker identification, we observe that our transcriptomics data analysis workflow was able to capture an immune‐related gene marker which is very recently shown to have a significant function in the determination of M1 macrophage polarisation in Inflammatory Bowel Disease (IBD).^40^ Although our analysis on immune marker identification shows significant changes in macrophage abundances in the data sets, experimental validations of the effect of ECM1 gene on macrophages in HCC TME settings are required. In their review, Hallman et al., also point out the same need to fully understand the regulation of immune cell migration and trafficking by ECM.^42^

A recent benchmark study on the deconvolution methods' capabilities and limitations were conducted based on a single‐cell RNA‐seq dataset of ∼11,000 cells from the TME in order to simulate bulk samples of known cell‐type proportions.^43^ This study revealed that, in most of the cases, deconvolution algorithms are prone to suffer from background predictions resulting in cell‐type predictions even in their absence. The authors further demonstrated that EPIC has a low background prediction for CAFs and NK cells, and quanTIseq has a low background prediction for T CD4^+^ cells and NK cells.^43^ These differences in the background predictions might account for the inconsistent results obtained in our study for monocytes, CAFs, NK cell, T CD4^+^ cell, dendritic cell (DC), and B cell. The quanTIseq algorithm is specifically developed for RNA‐seq data, but also provides optimised mode for microarray datasets. In our analysis, the optimised mode was used by considering the potential cost of accuracy. EPIC was not able to estimate mRNA content for CAFs and endothelial cells. Thus, estimated scores were not considered as absolute cell proportions rather as absolute scores of cell type contents. Furthermore, the CIBERSORT based cell type comparison was conducted considering 48 significantly deconvoluted samples out of 511 HCCDB3 samples forming a further limitation of our study resulting in a lower statistical power for this particular dataset. As the final limitation of our study, we would like to note that different array platforms employed in this study might have a potential effect on the biological significance of our results.

## 7 CONCLUSIONS

In this study, we identified ECM1 gene as a potential immune‐related marker acting through immune cell migration and macrophage polarisation. Moreover, our results indicate that macrophages, such as M0 macrophage and M1 macrophage cells, undergo significant changes in HCC TME. Therefore, genes that have crucial functions in their behavior, such as the ECM1 gene, may be suitable novel candidate targets that can be exploited so as to gain a better understanding of their role in HCC and provide potential therapeutics^44^ insight.

## AUTHORS' CONTRIBUTIONS

All authors have made a substantial, direct intellectual contribution to this study. Conceptualisation, Animesh Acharjee; methodology and investigation Basak Bahcivanci, Animesh Acharjee; writing original draft preparation, Basak Bahcivanci; writing review and editing, Roshan Shafiha, Basak Bahcivanci, Georgios V. Gkoutos, Animesh Acharjee; supervision, Georgios V. Gkoutos, Animesh Acharjee; project administration, Basak Bahcivanci, Animesh Acharjee; All authors have read and agreed to the published version of the manuscript.

## CONFLICT OF INTEREST

The authors declare no conflict of interest.

## FUNDING INFORMATION

Animesh Acharjeeand Georgios V. Gkoutosacknowledge support from the NIHR Birmingham ECMC, NIHR Birmingham SRMRC, Nanocommons H2020‐EU (731032) and the NIHR Birmingham Biomedical Research Centre, and the MRC Health Data Research UK (HDRUK/CFC/01), an initiative funded by UK Research and Innovation, Department of Health and Social Care (England) and the devolved administrations, and leading medical research charities. The views expressed in this publication are those of the authors and not necessarily those of the NHS, the National Institute for Health Research, the Medical Research Council or the Department of Health.

## AVAILABILITY OF DATA AND MATERIALS

All the data used in this study are freely and publicly available. They can be accessed at http://lifeome.net/database/hccdb/download.html. All codes are available at https://github.com/basakbahcivanci/UoB‐HCC.git.

## Supporting information


Appendix S1

## Data Availability

Data sharing is not applicable to this article as no new data were created or analyzed in this study.
