# Supplementary material for: Associating transcriptomics data with inflammatory markers to understand tumour microenvironment in hepatocellular carcinoma
Source: Cancer Med. 2022 Jun 18;12(1):696–711. doi: 10.1002/cam4.4941 (PMC9844659; doi:10.1002/cam4.4941)
Supplement: Supplementary file 1 — Appendix S1 [file CAM4-12-696-s001.zip › cam44941-sup-0001-TableS1.docx]

|  | **Table S1 Overview of deconvolution methods** | | | | | | |  |
| --- | --- | --- | --- | --- | --- | --- | --- | --- |
|  | **Tool** | **Approach** | **Score** | **Comparisons** | **Cell types** | **Reference** | | |
|  | MCP-counter | Enrichment Based | Absolute scores | Inter | 8 immune cell types, fibroblasts, endothelial cells | | [14] |  |
|  | CIBERSORT | ν-SVR algorithm | Absolute scores,  relative to the total immune cells in a sample | Inter | 22 immune cell types | | [25] |  |
|  | TIMER | constrained least squares regression | Absolute scores | Inter | 6 immune cell types | | [28] |  |
|  | EPIC | constrained least squares regression | Absolute scores, relative to all cell types in a sample | Intra, inter | 6 immune cell types, fibroblasts, endothelial cells | | [29] |  |
|  | quanTIseq | constrained least squares regression | Cell fractions,  relative to all cell types in a sample | Intra, inter | 10 immune cell types | | [31] |  |
|  | Inter: Inter-sample comparison. Intra: Intra-sample comparison. | | | | | | |  |
