# Supplementary material for: Associating transcriptomics data with inflammatory markers to understand tumour microenvironment in hepatocellular carcinoma
Source: Cancer Med. 2022 Jun 18;12(1):696–711. doi: 10.1002/cam4.4941 (PMC9844659; doi:10.1002/cam4.4941)
Supplement: Supplementary file 1 — Appendix S1 [file CAM4-12-696-s001.zip › cam44941-sup-0002-FigureS1.pdf]

Supplementary Figures

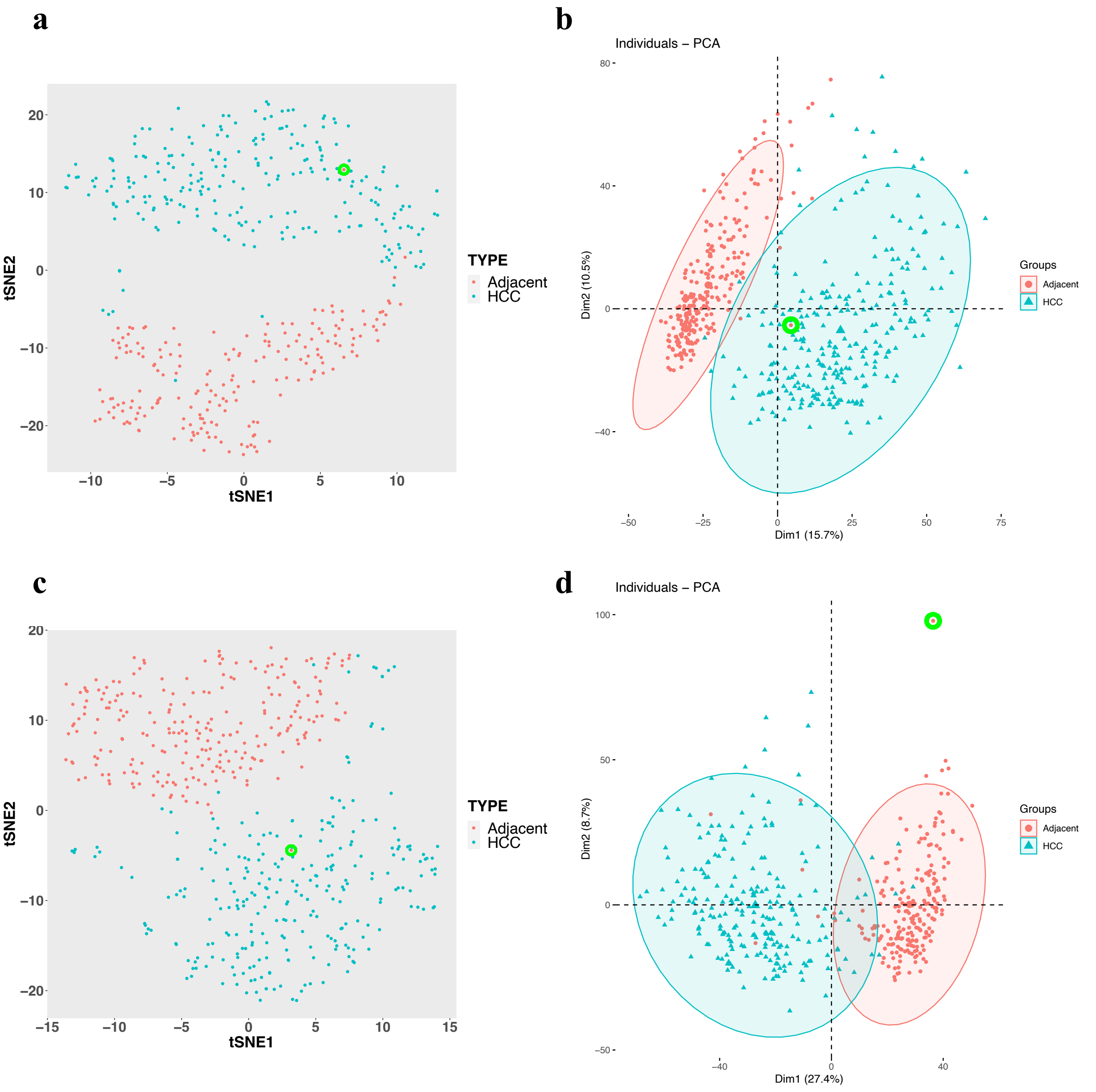

Figure S1: **a** t-SNE plot of HCCDB4 dataset with adjacent Sample 121 in the green circle. **b** PCA plot of HCCDB3 dataset. **c** t-SNE plot of HCCDB3 dataset. **d** t-SNE plot of HCCDB6 dataset. **d** PCA plot of HCCDB6 dataset.

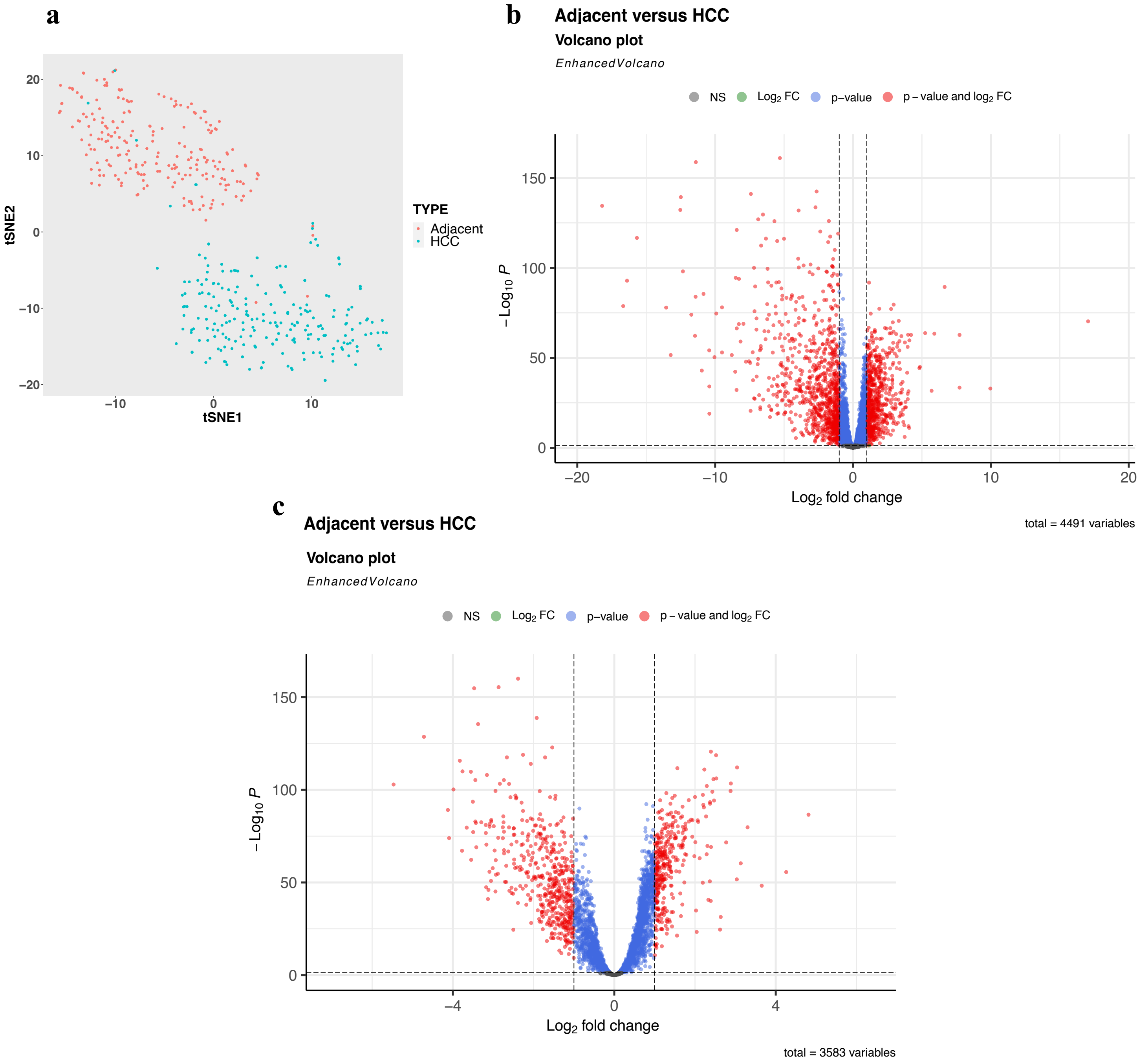

Figure S2: **a** t-SNE plot of HCCDB6 dataset. **b** Volcano plot of HCCDB3 dataset. **c** Volcano plot of HCCDB6 dataset.

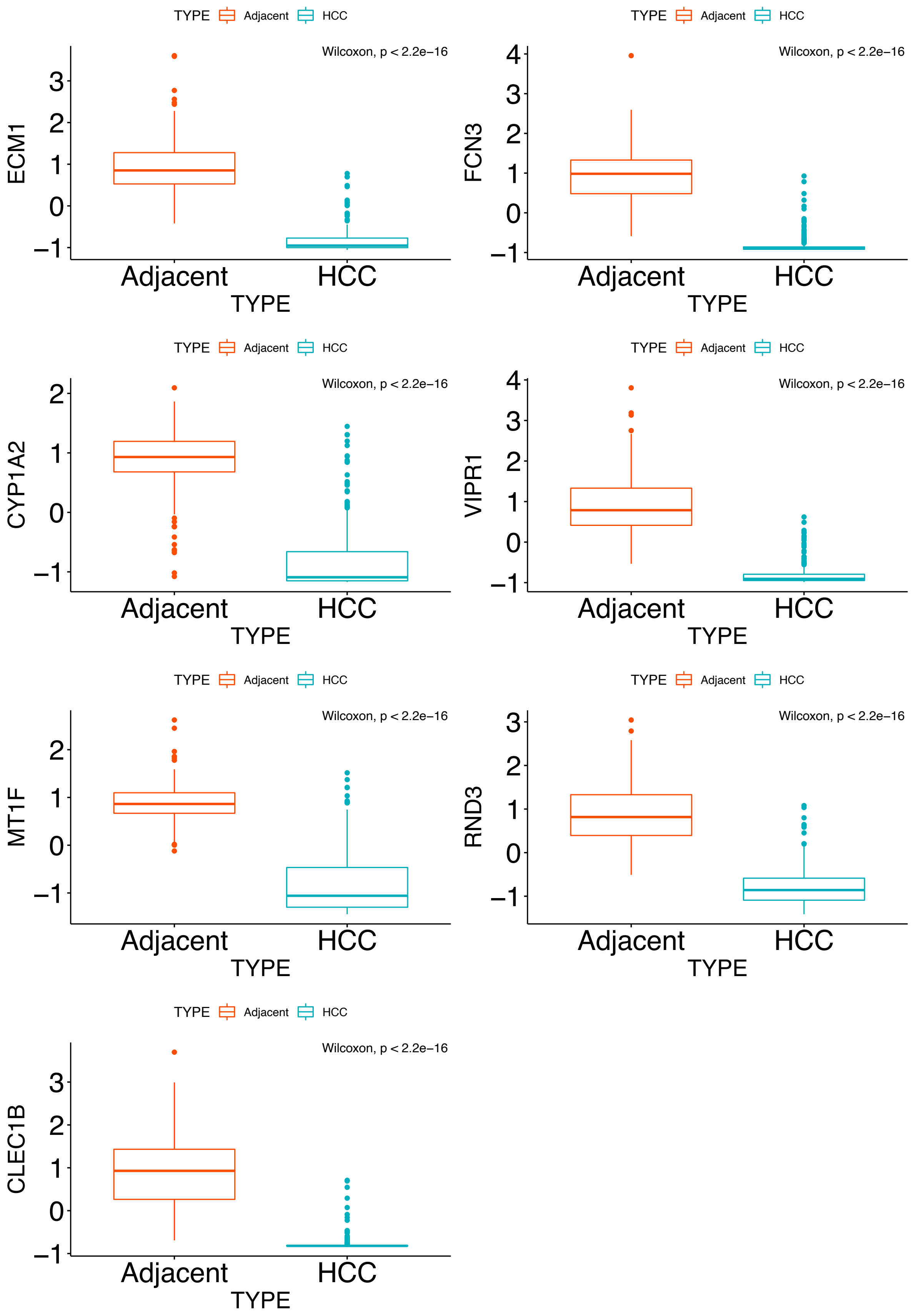

Figure S3: Down regulated genes in HCCDB3 data.

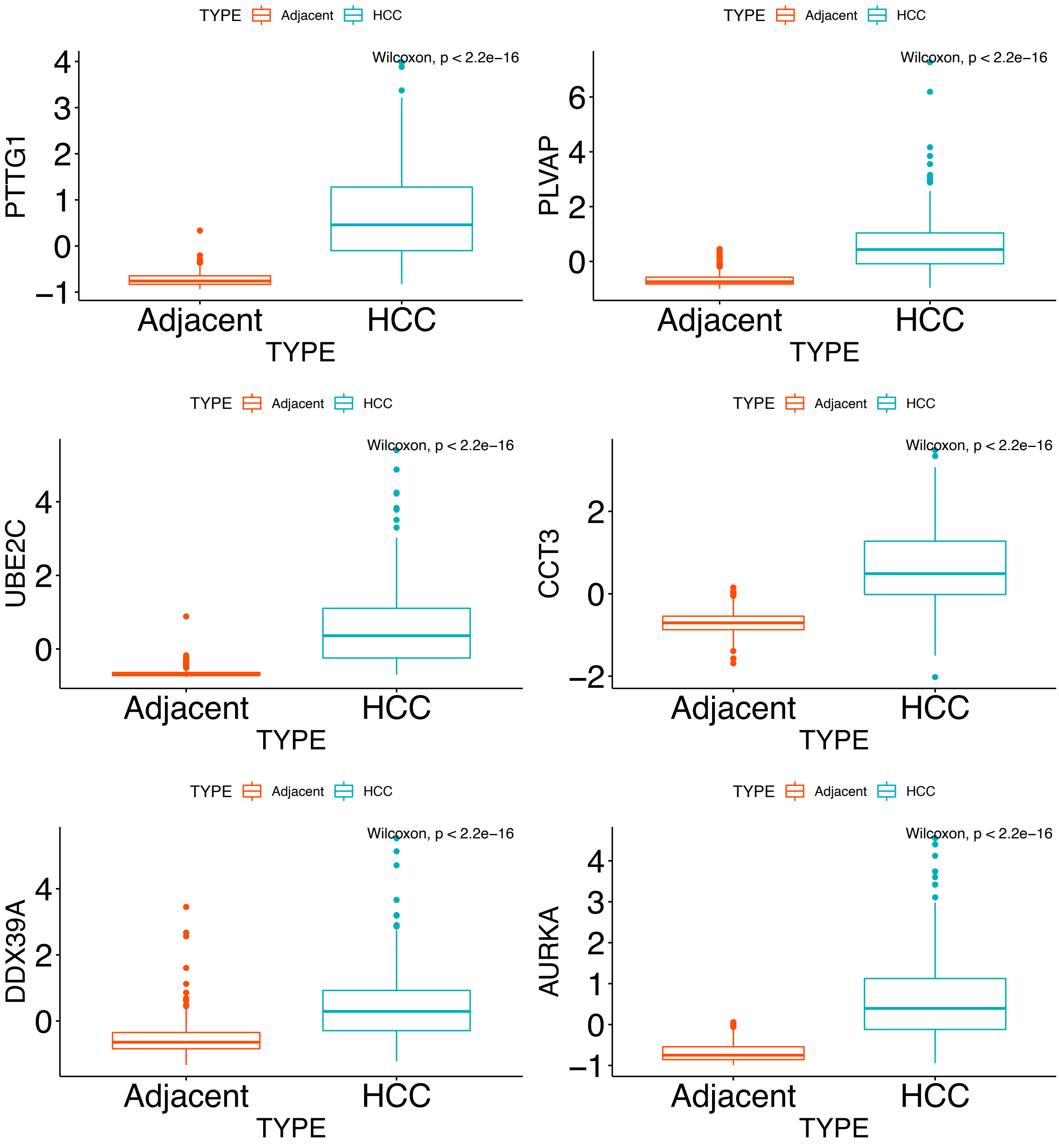

Figure S4: Up regulated genes in HCCDB3 data.

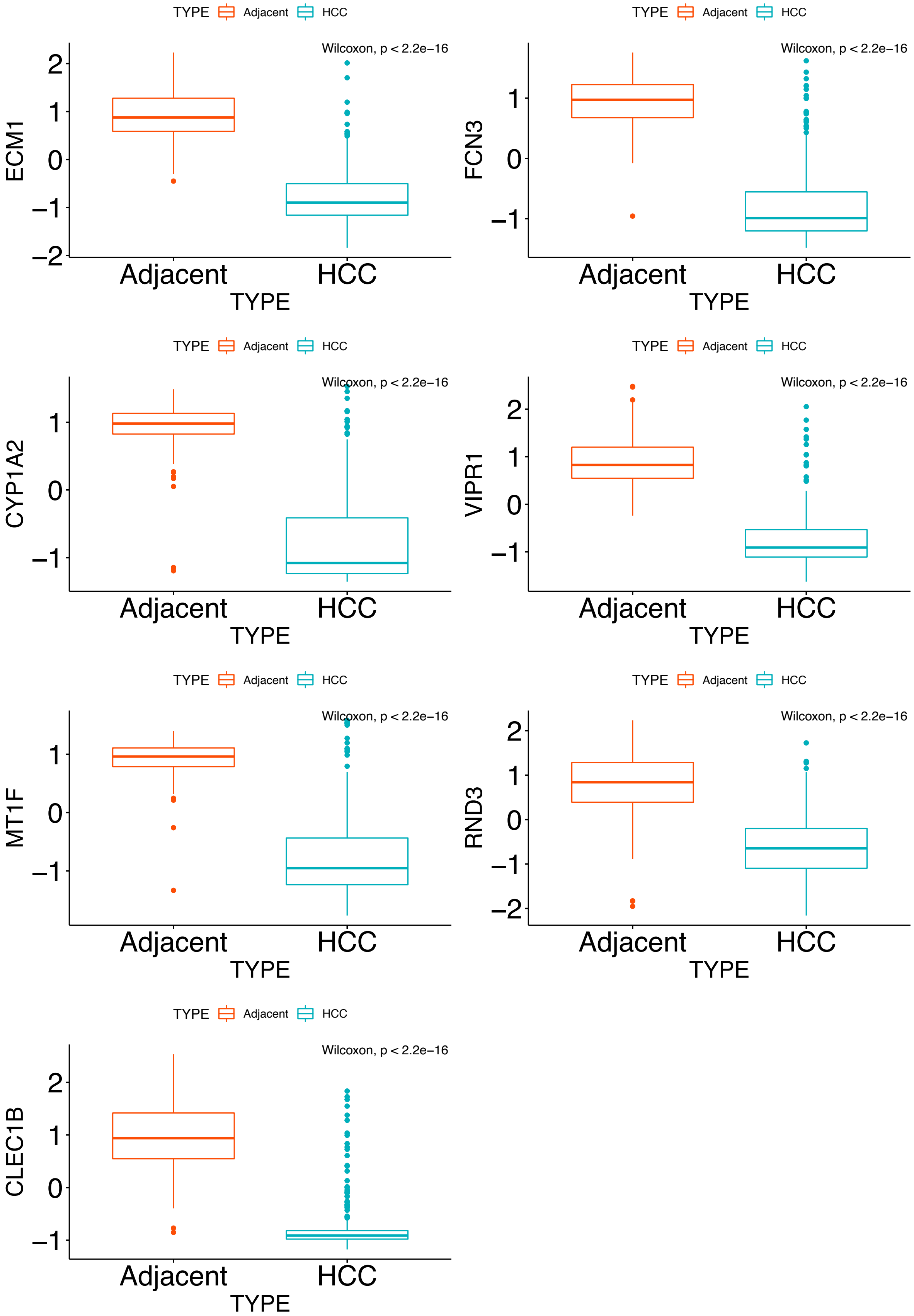

Figure S5: Down regulated genes in HCCDB4 data.

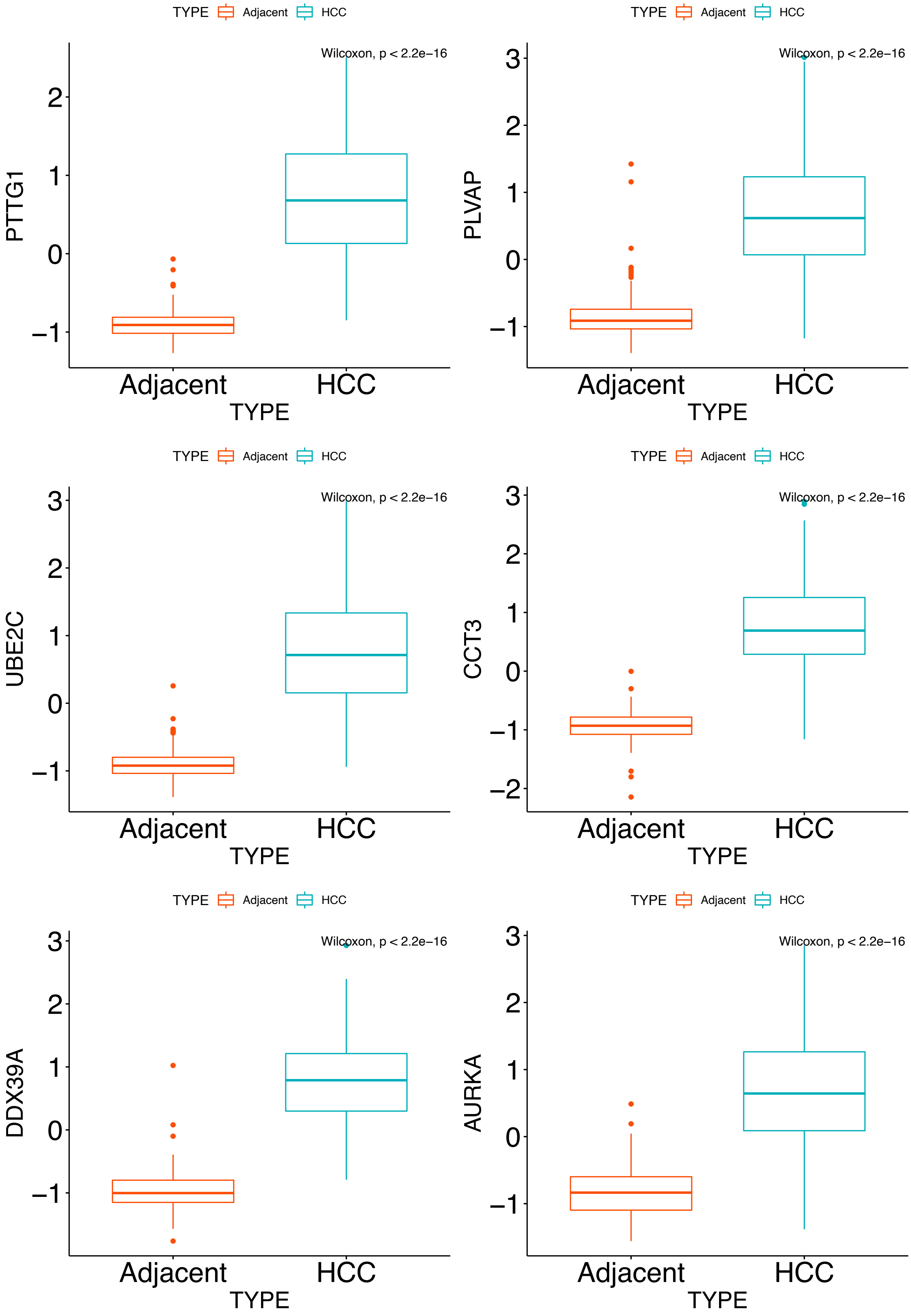

Figure S6: Up regulated genes in HCCDB4 data.

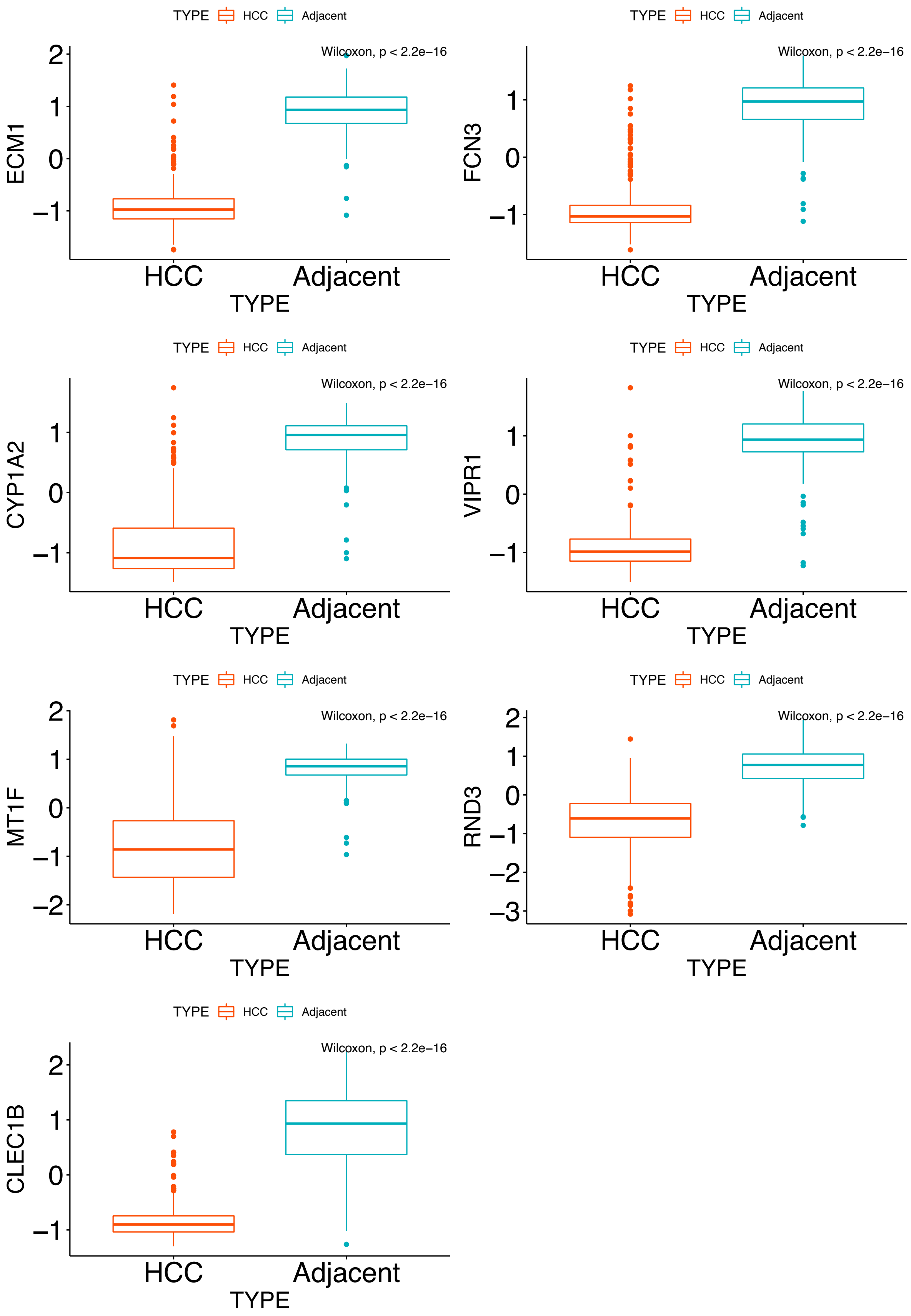

Figure S7: Down regulated genes in HCCDB6 data.

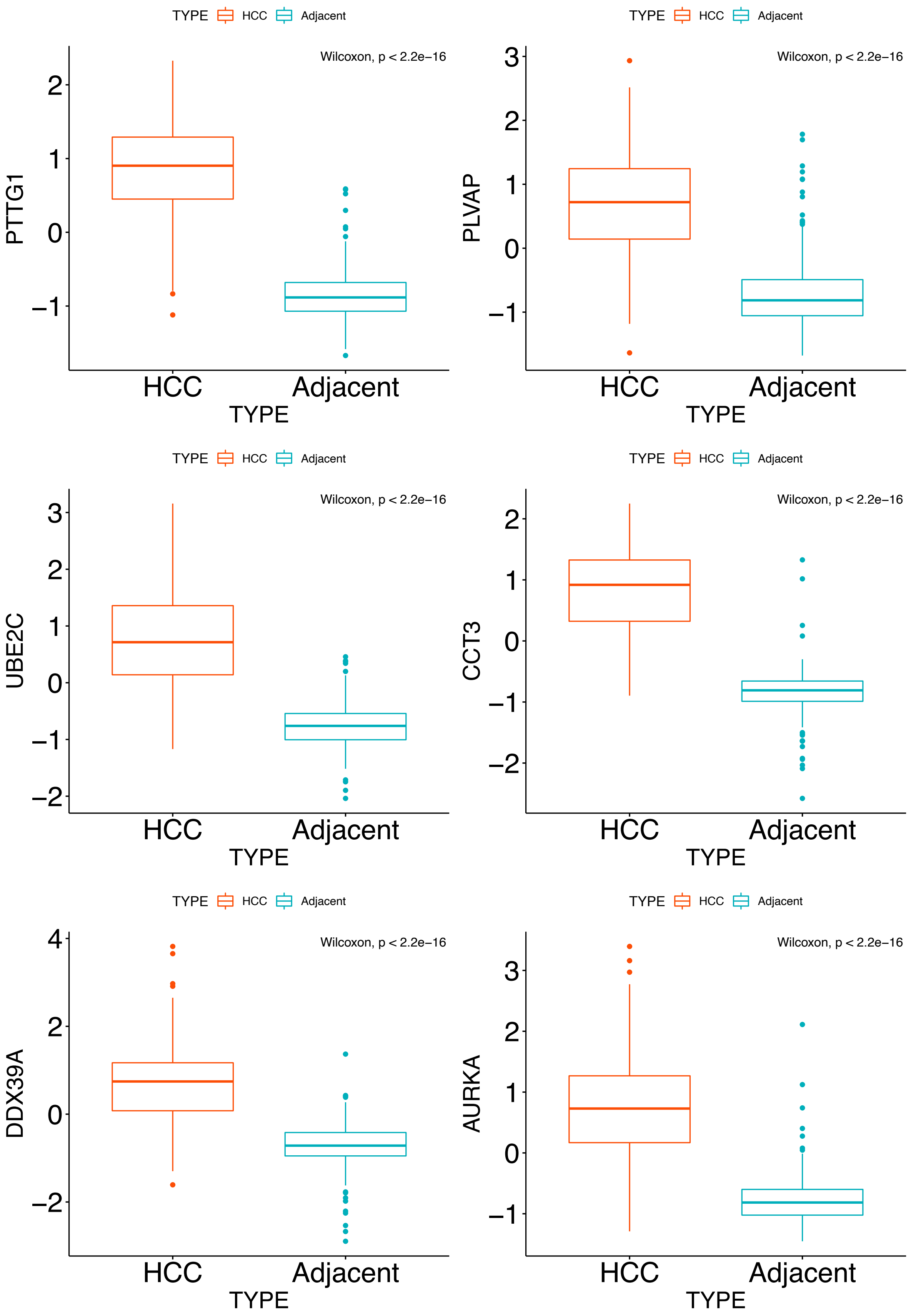

Figure S8: Up regulated genes in HCCDB6 data.

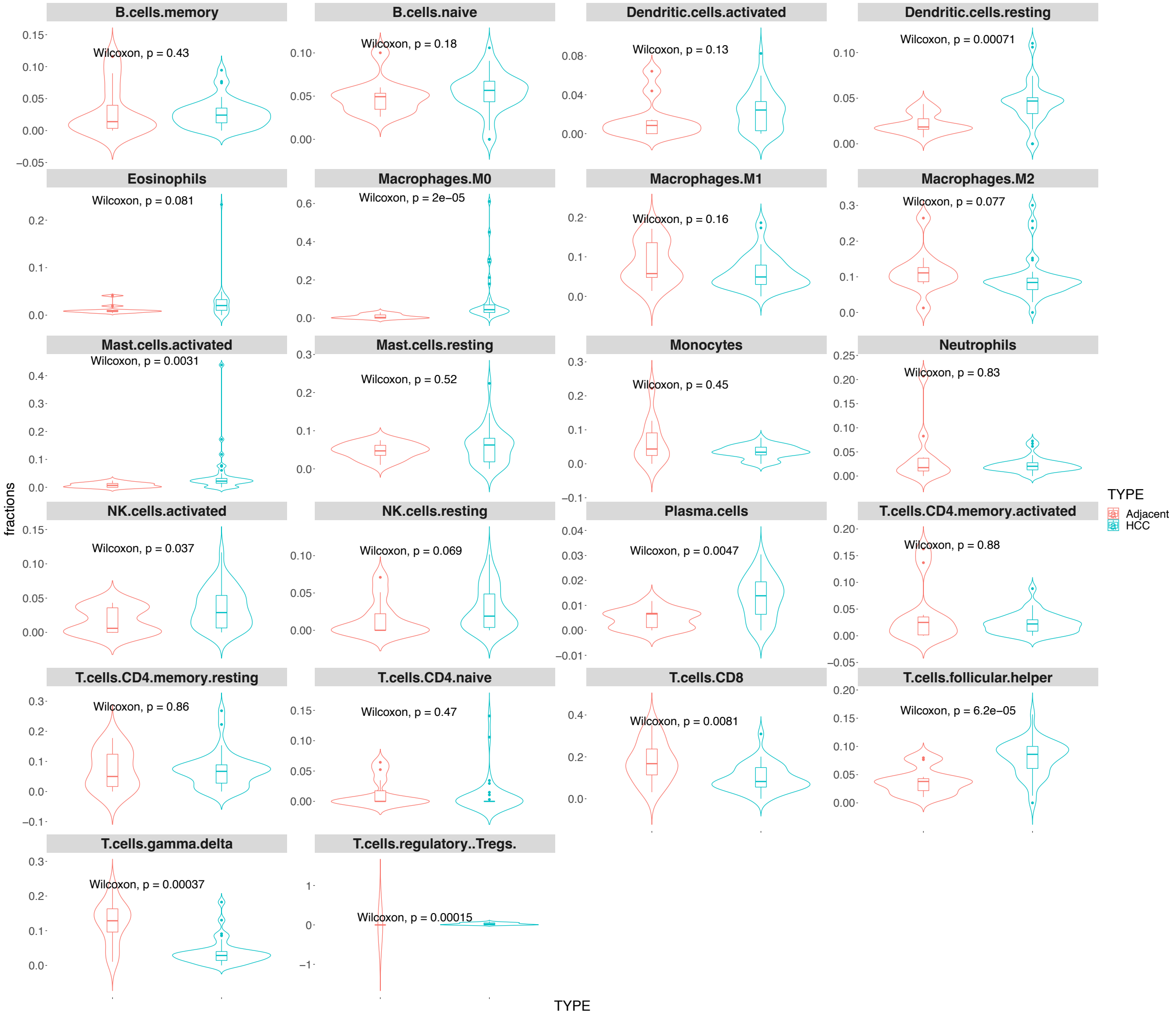

Figure S9: HCCDB3 dataset. Violin box plots of CIBERSORT show abundance scores of cell type fractions for adjacent and tumour (HCC) groups in HCCDB3 dataset. Two-sided Wilcoxon rank-sum tests were performed to compare two groups for each cell type with 0.05 significance level. 48 significantly deconvoluted samples out of 511 samples are considered when comparing groups. (11 out of 48 samples are adjacent.)

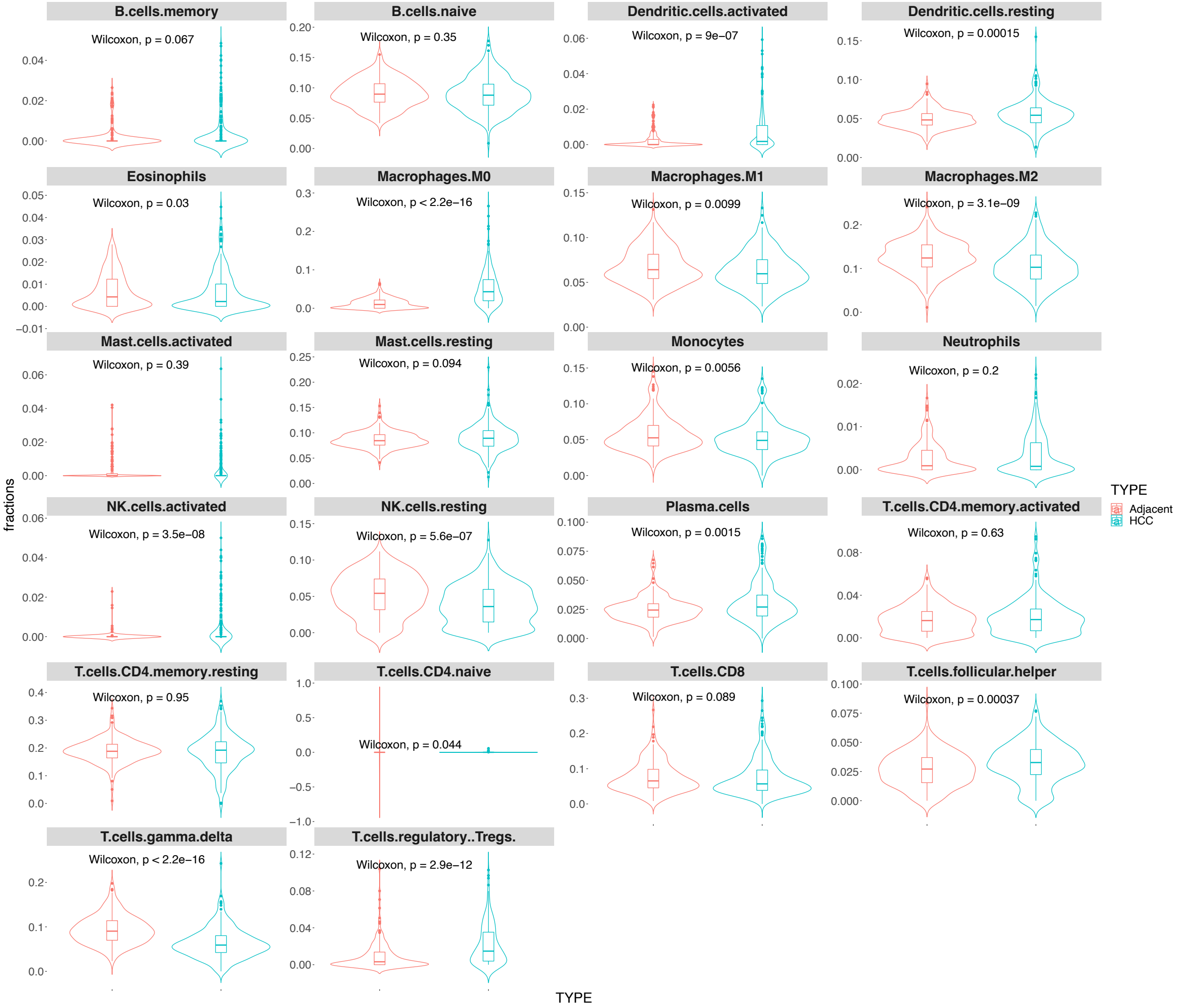

Figure S10: CIBERSORT cell type abundance comparison between adjacent and tumour groups in HCCDB4 dataset. Violin box plots of CIBERSORT show abundance scores of cell type fractions for adjacent and tumour (HCC) groups in HCCDB4 dataset. Two-sided Wilcoxon rank-sum tests were performed to compare two groups for each cell type with 0.05 significance level. Only one sample was found to be insignificant with CIBERSORT

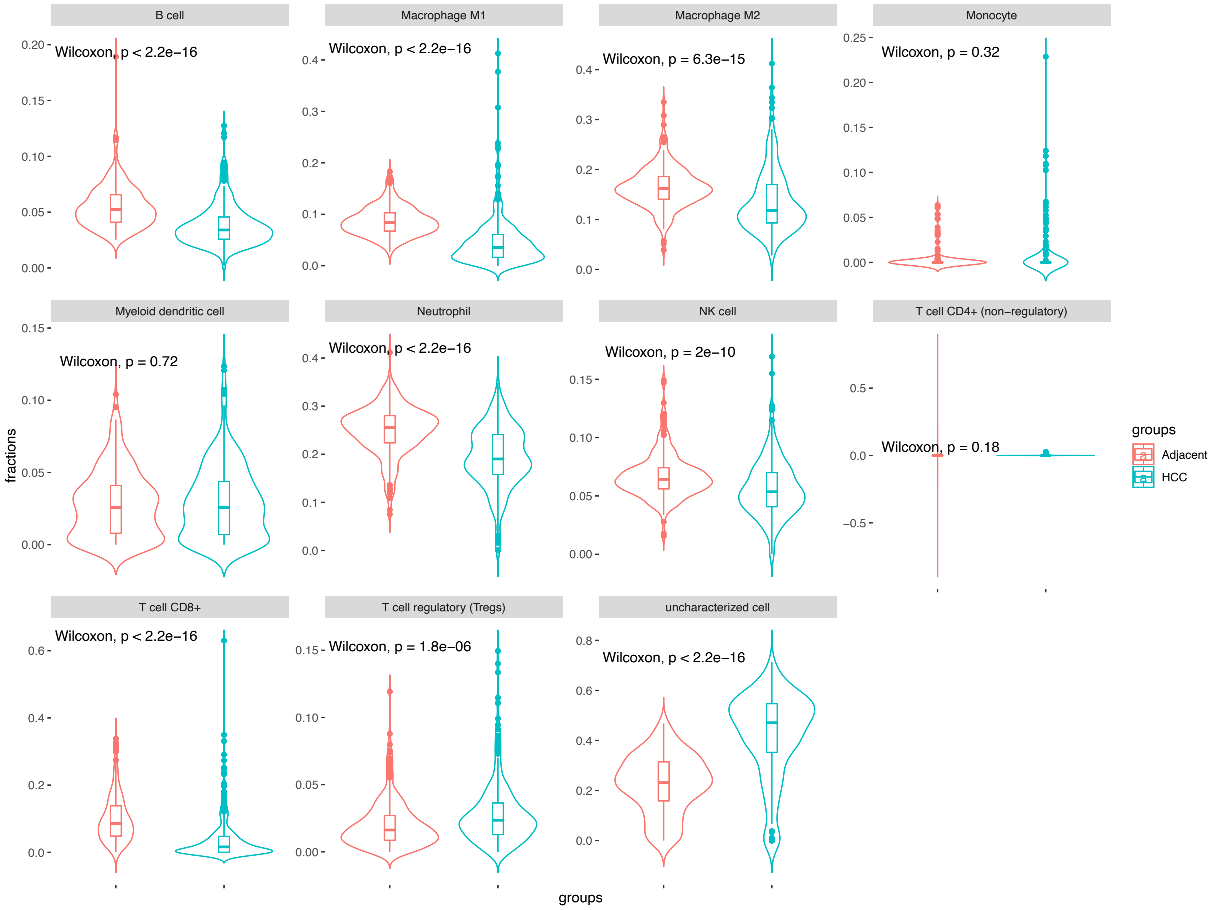

Figure S11: quanTIseq cell type fraction comparison between adjacent and tumour samples in HCCDB3 dataset. Violin box plots of quanTIseq show cell type fractions for adjacent and tumour (HCC) groups in HCCDB3 dataset. Two-sided Wilcoxon rank-sum tests were performed to compare two groups for each cell type with 0.05 significance level.

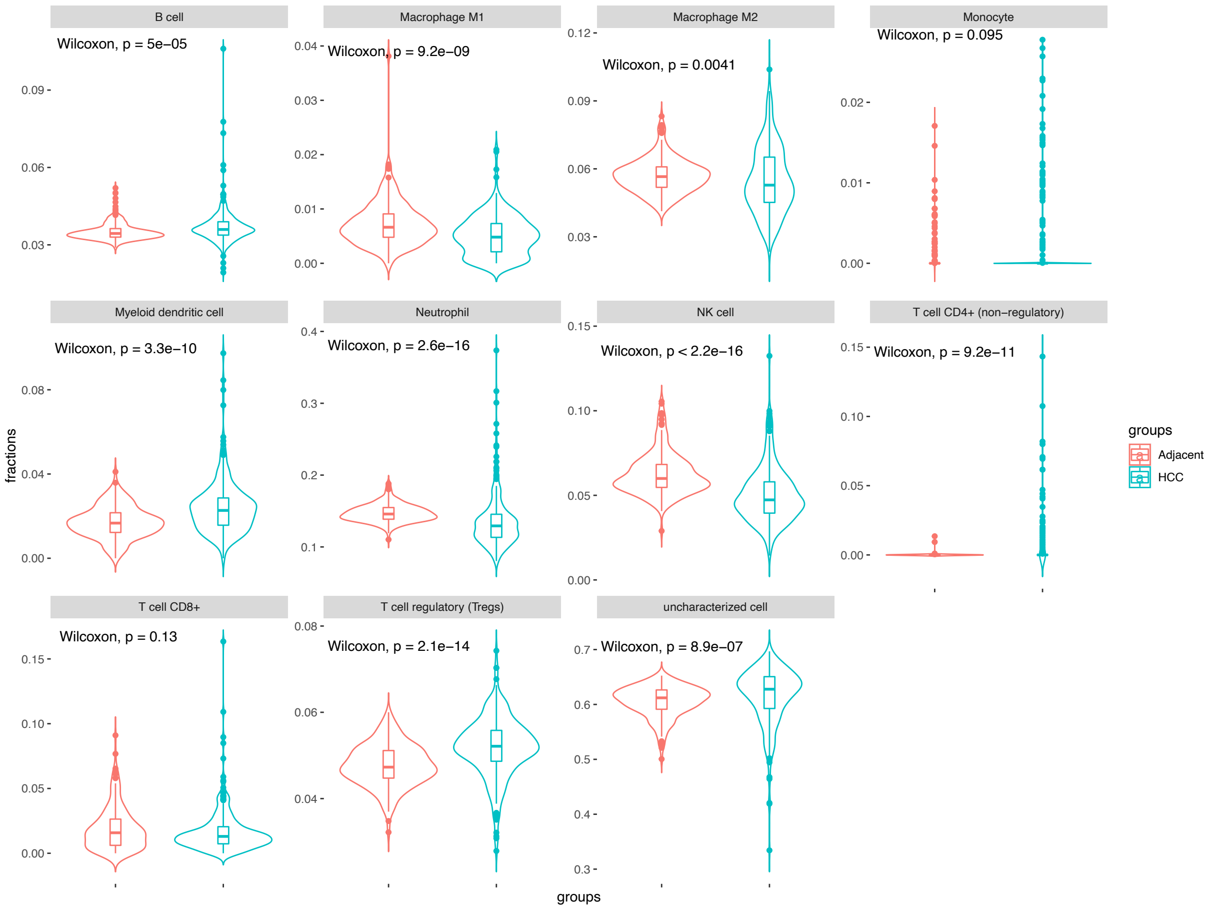

Figure S12: quanTIseq cell type fraction comparison between adjacent and tumour samples in HCCDB4 dataset. Violin box plots of quanTIseq show cell type fractions for adjacent and tumour (HCC) groups in HCCDB4 dataset. Two-sided Wilcoxon rank-sum tests were performed to compare two groups for each cell type with 0.05 significance level.

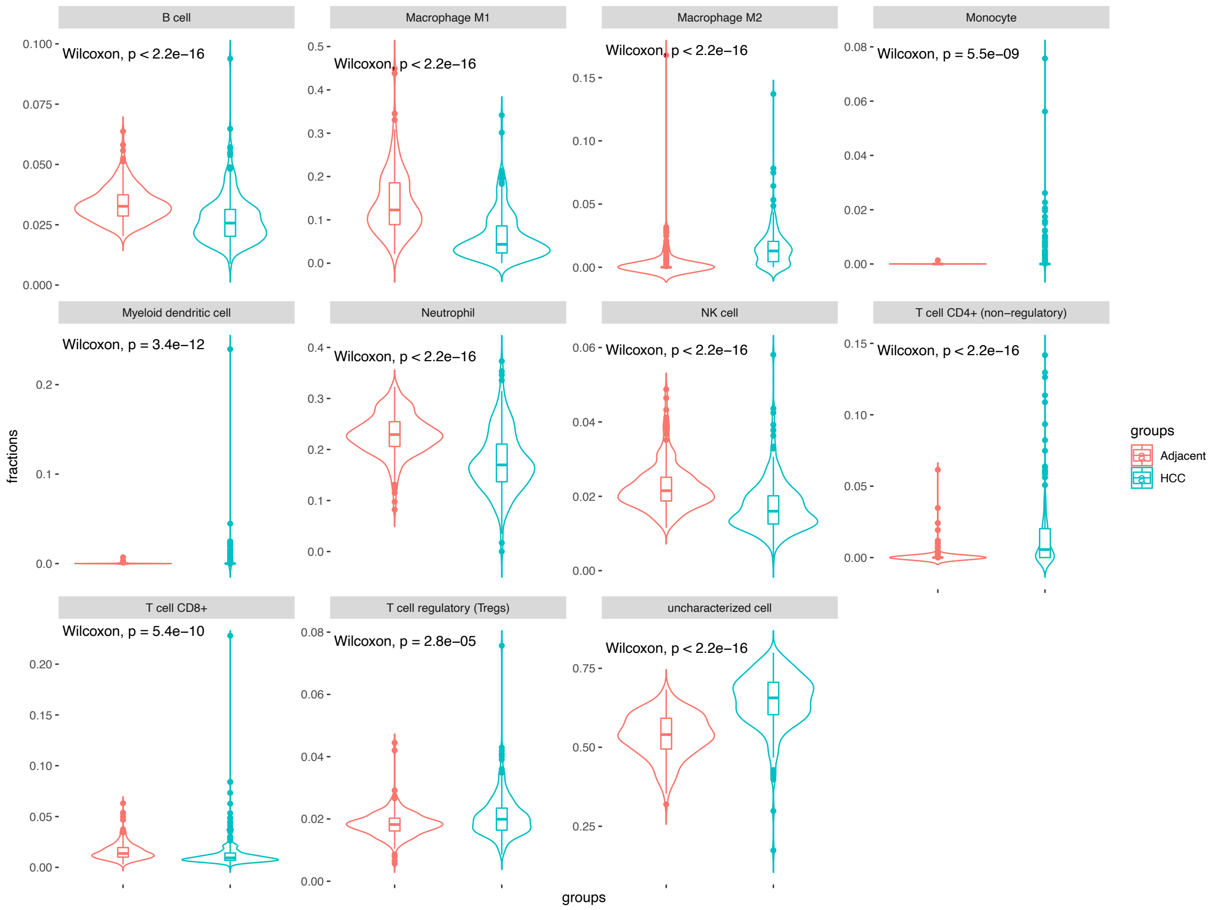

Figure S13: quanTIseq cell type fraction comparison between adjacent and tumour samples in HCCDB6 dataset. Violin box plots of quanTIseq shows cell type fractions for adjacent and tumour (HCC) groups in HCCDB6 dataset. Two-sided Wilcoxon rank-sum tests were performed to compare two groups for each cell type with 0.05 significance level.

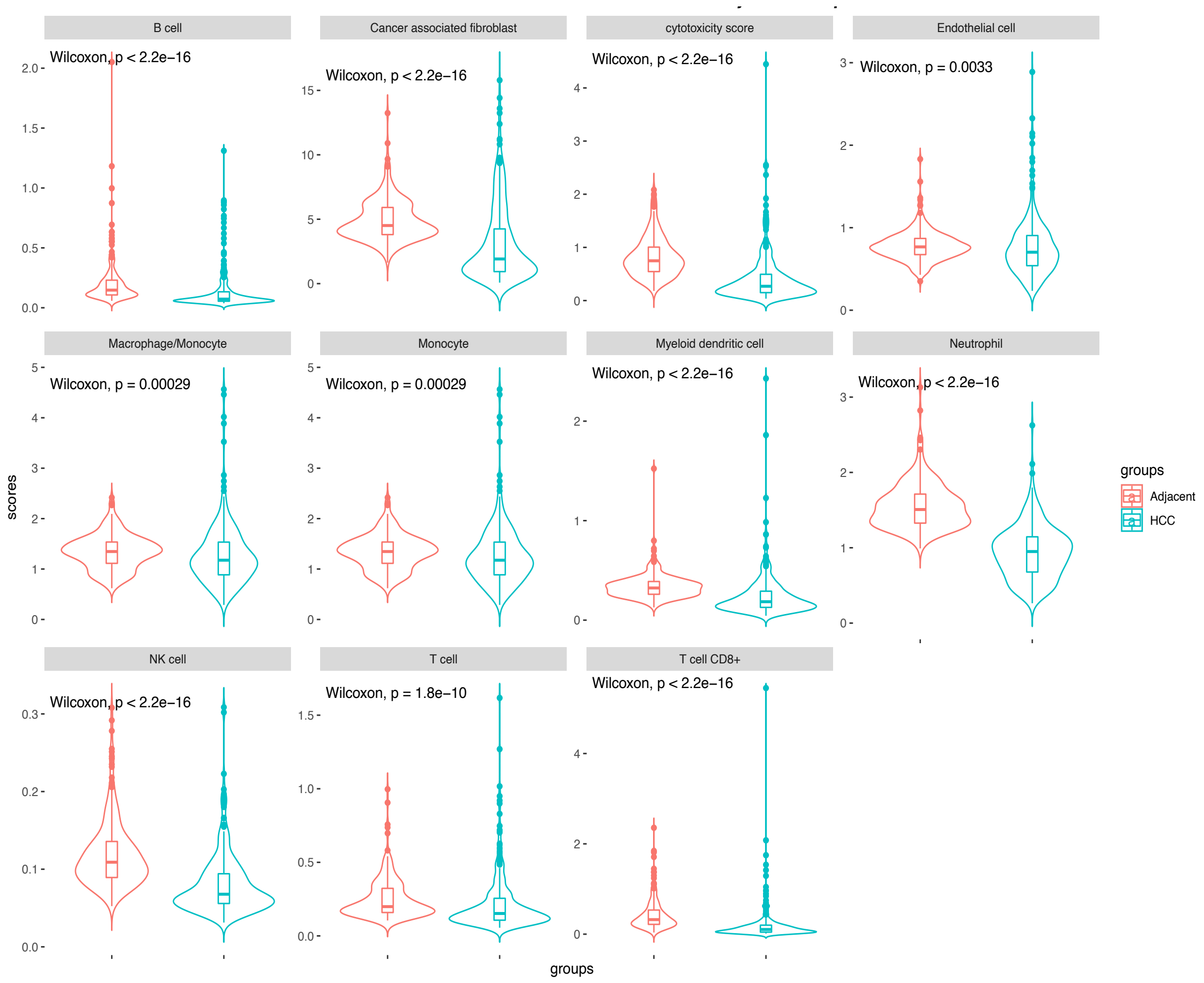

Figure S14: Violin plot of MCP-counter algorithm for HCCDB3. Violin boxs plot of MCP-counter show estimated abundance scores of cell types for adjacent and tumour (HCC) groups in HCCDB3 dataset. Two-sided Wilcoxon rank-sum tests were performed to compare two groups for each cell type with 0.05 significance level.

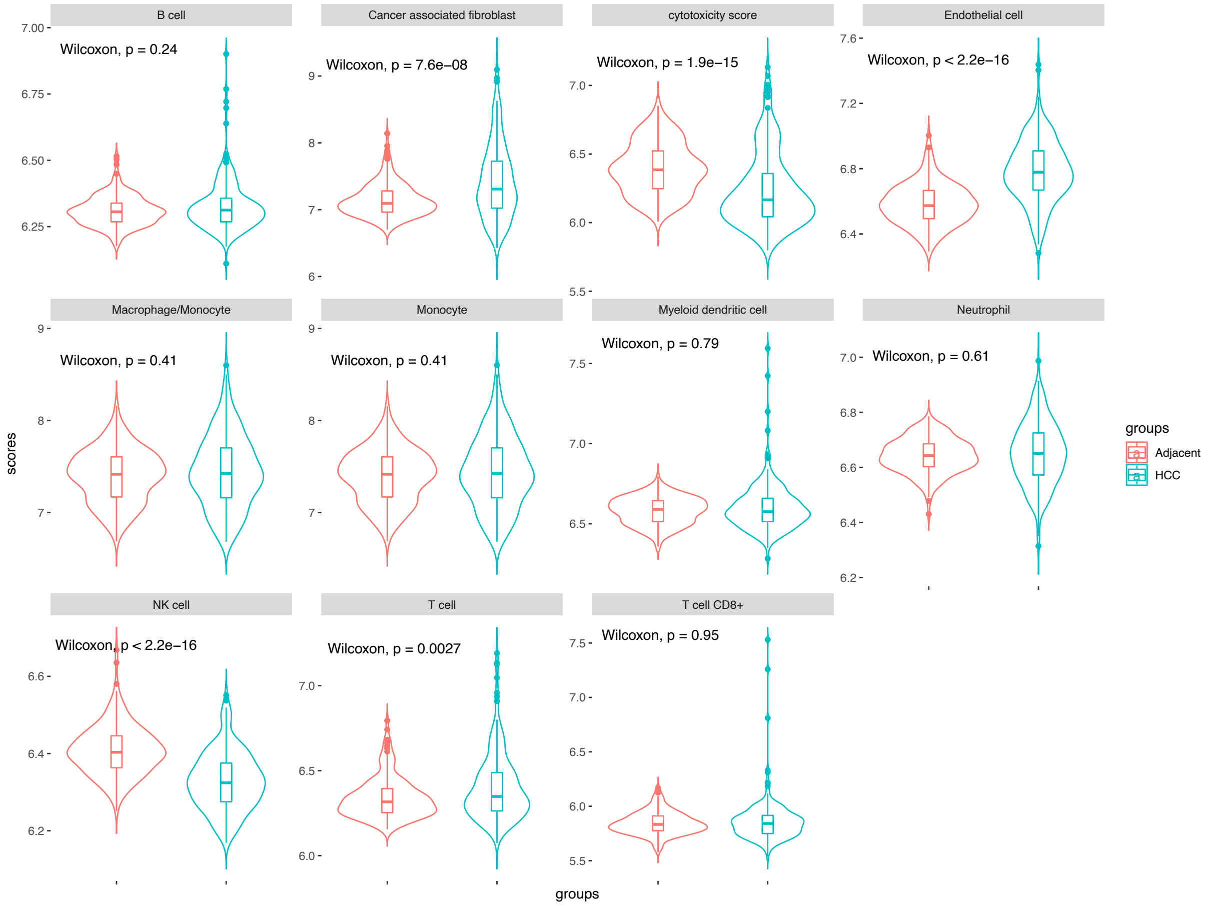

Figure S15: Violin plot of MCP-counter algorithm for HCCDB4. Violin box plot of MCP-counter shows estimated abundance scores of cell types for adjacent and tumour (HCC) groups in HCCDB4 dataset. Two-sided Wilcoxon rank-sum tests were performed to compare two groups for each cell type with 0.05 significance level.

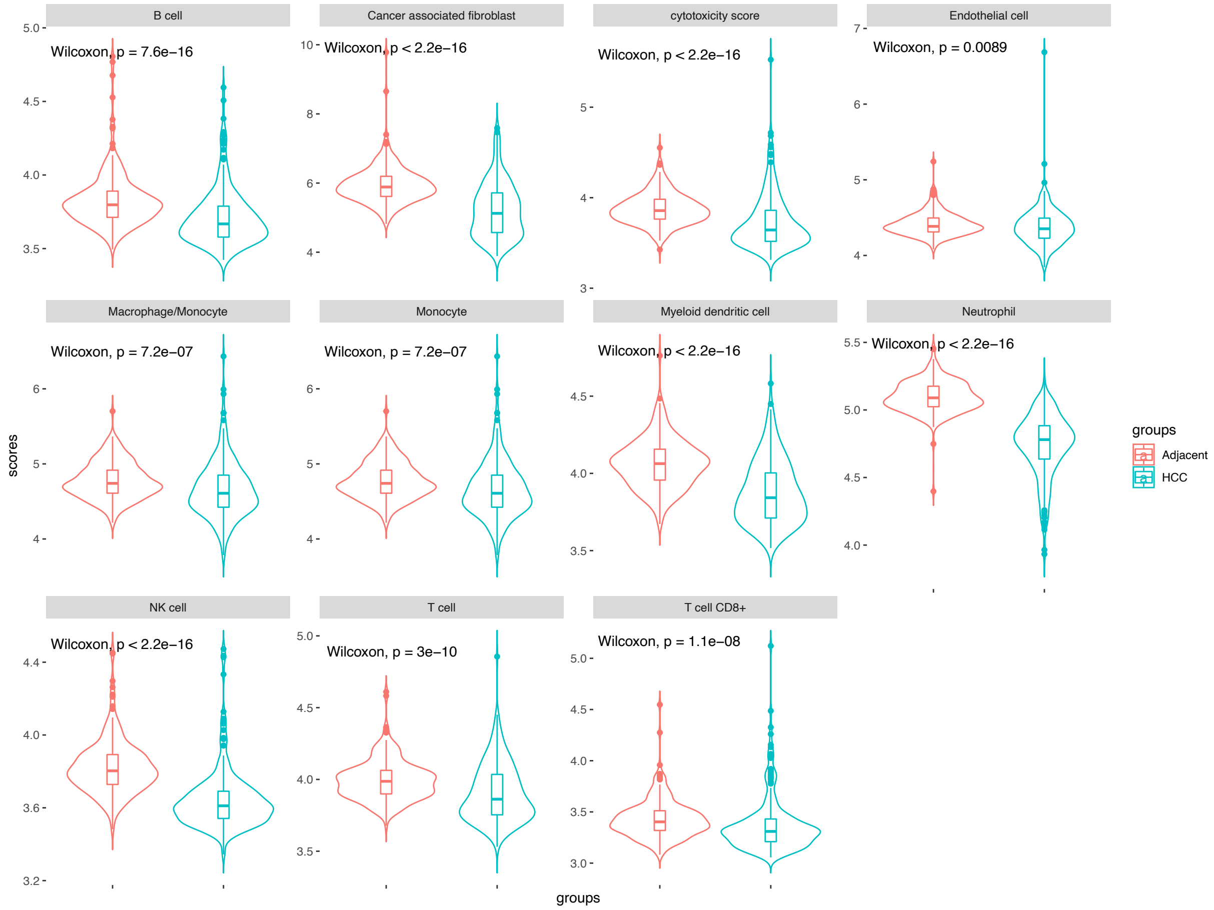

Figure S16: Violin plot of MCP-counter algorithm for HCCDB6. Violin box plots of MCP-counter show abundance scores of cell types for adjacent and tumour (HCC) groups in HCCDB6 dataset. Two-sided Wilcoxon rank-sum tests were performed to compare two groups for each cell type with 0.05 significance level.

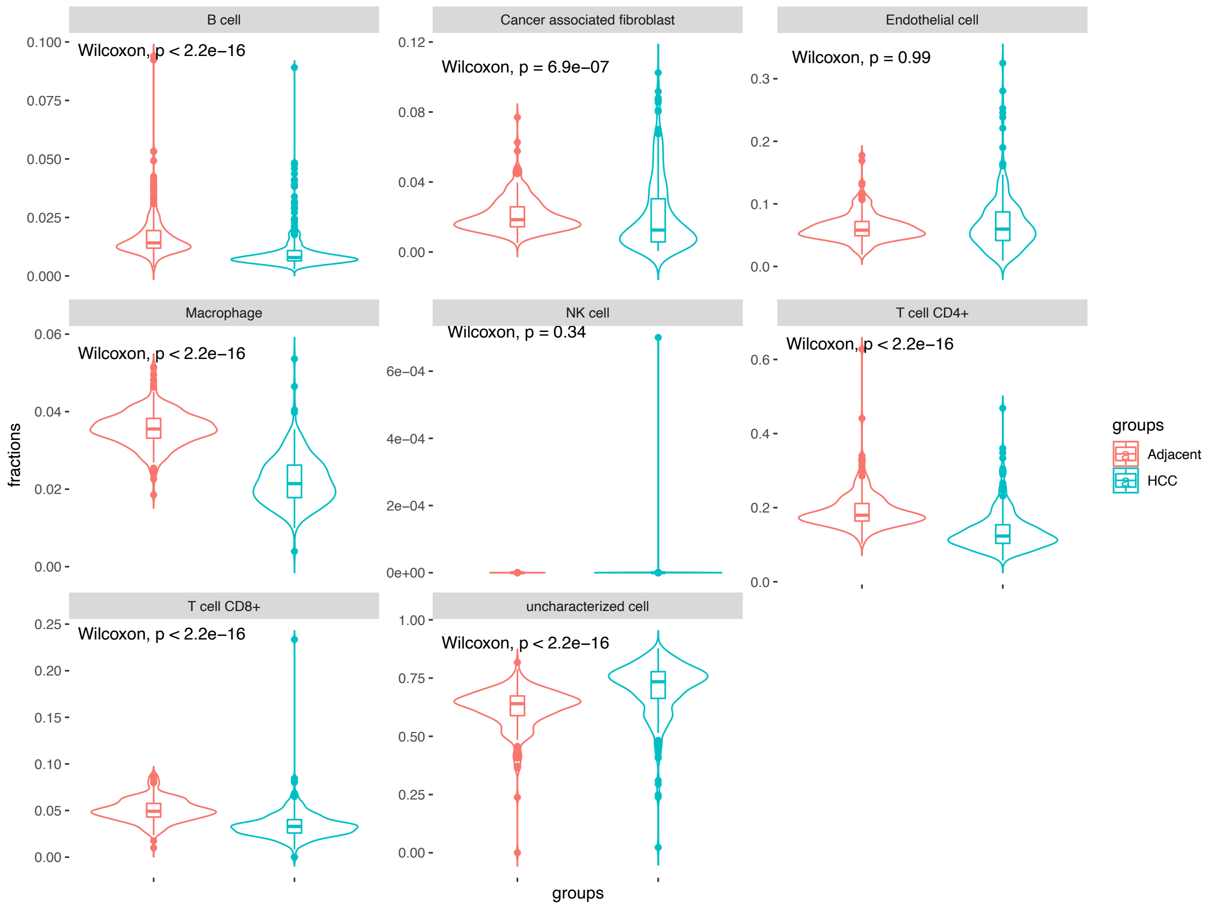

Figure S17: Estimation of cell type abundance between adjacent and tumour groups of HCCDB3 dataset by EPIC. Violin box plots of EPIC abundance scores of cell types for adjacent and tumour (HCC) groups in HCCDB3 dataset. Two-sided Wilcoxon rank-sum tests were performed to compare two groups for each cell type with 0.05 significance level.

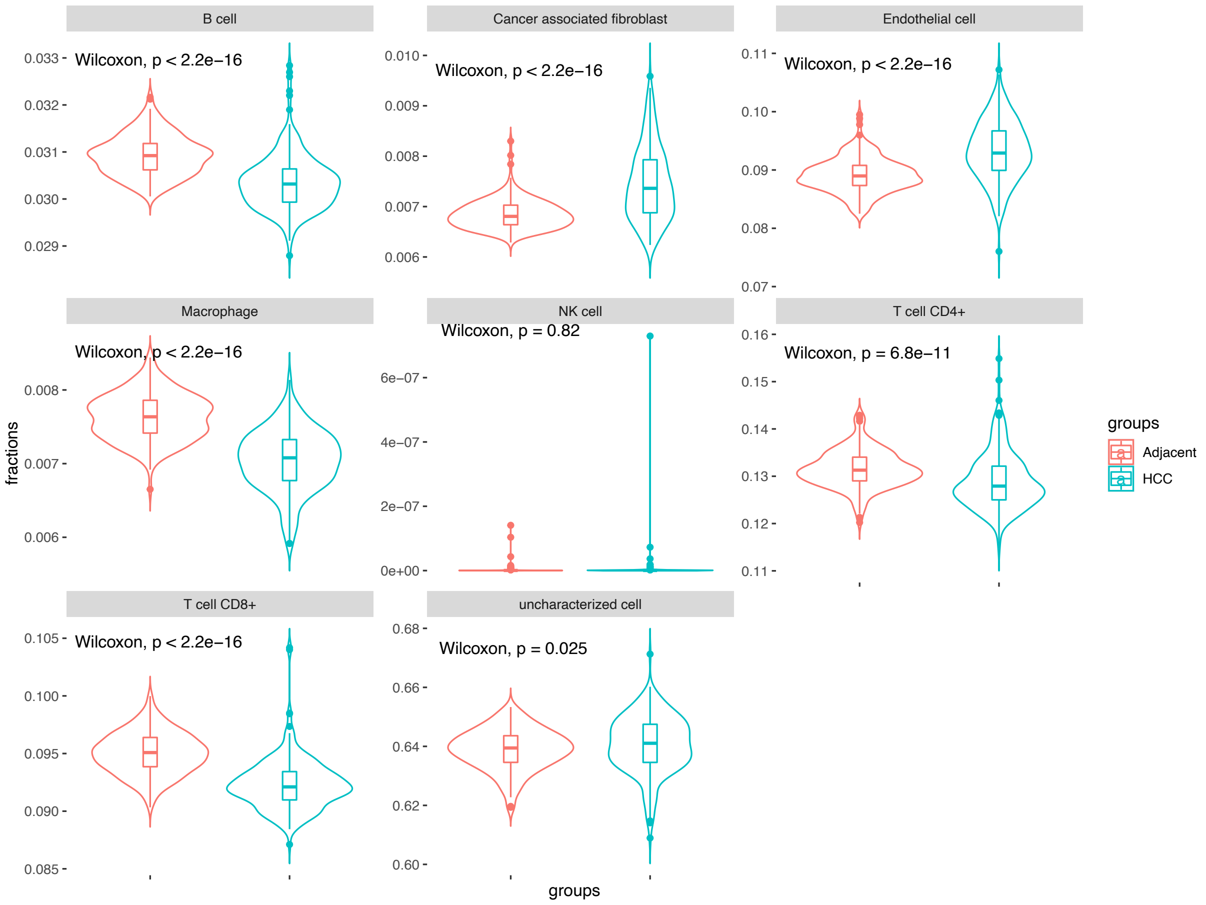

Figure S18: Estimation of cell type abundance between adjacent and tumour groups of HCCDB4 dataset by EPIC. Violin box plots of EPIC show abundance scores of cell types for adjacent and tumour (HCC) groups in HCCDB4 dataset. Two-sided Wilcoxon rank-sum tests were performed to compare two groups for each cell type with 0.05 significance level.

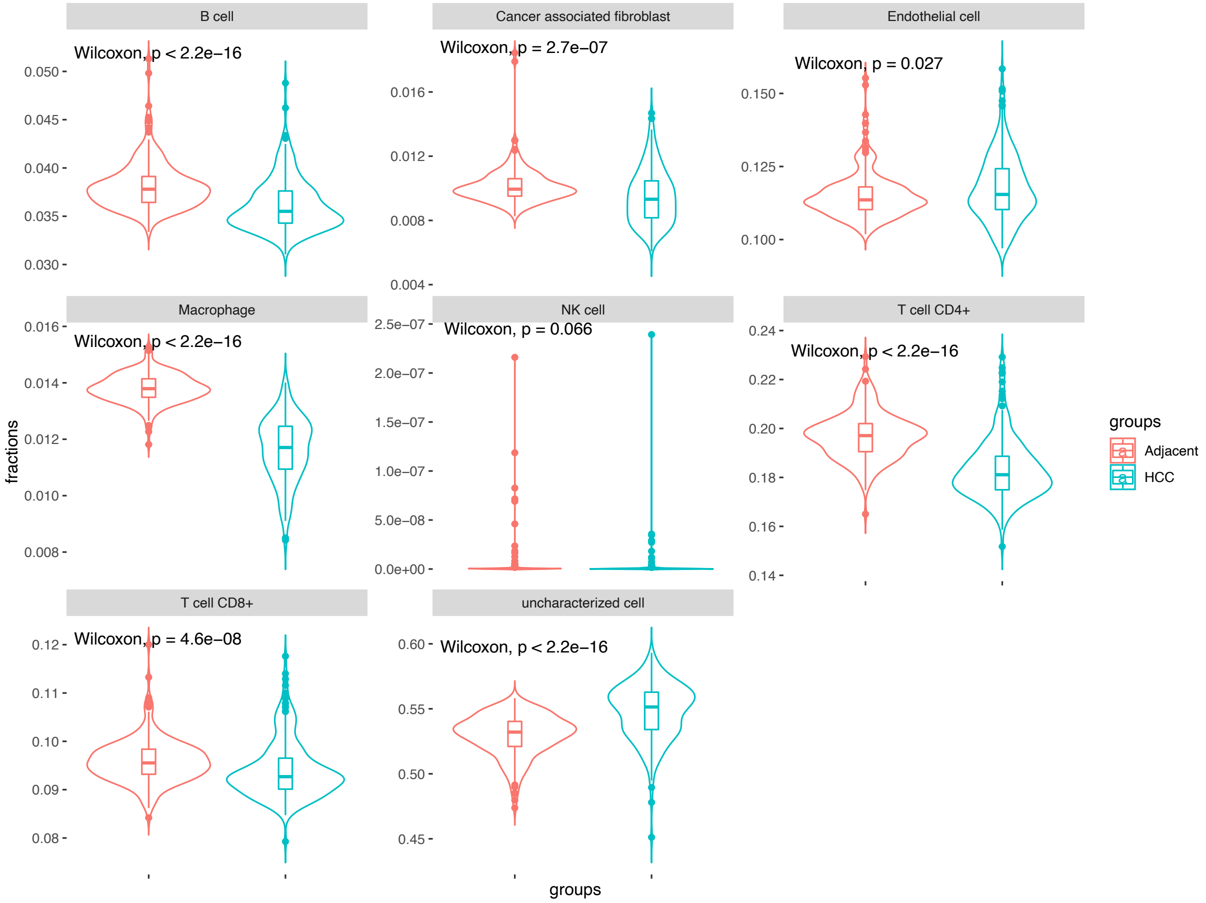

Figure S19: Estimation of cell type abundance between adjacent and tumour groups of HCCDB6 dataset by EPIC. Violin box plots of EPIC show abundance scores of cell types for adjacent and tumour (HCC) groups in HCCDB6 dataset. Two-sided Wilcoxon rank-sum tests were performed to compare two groups for each cell type with 0.05 significance level.

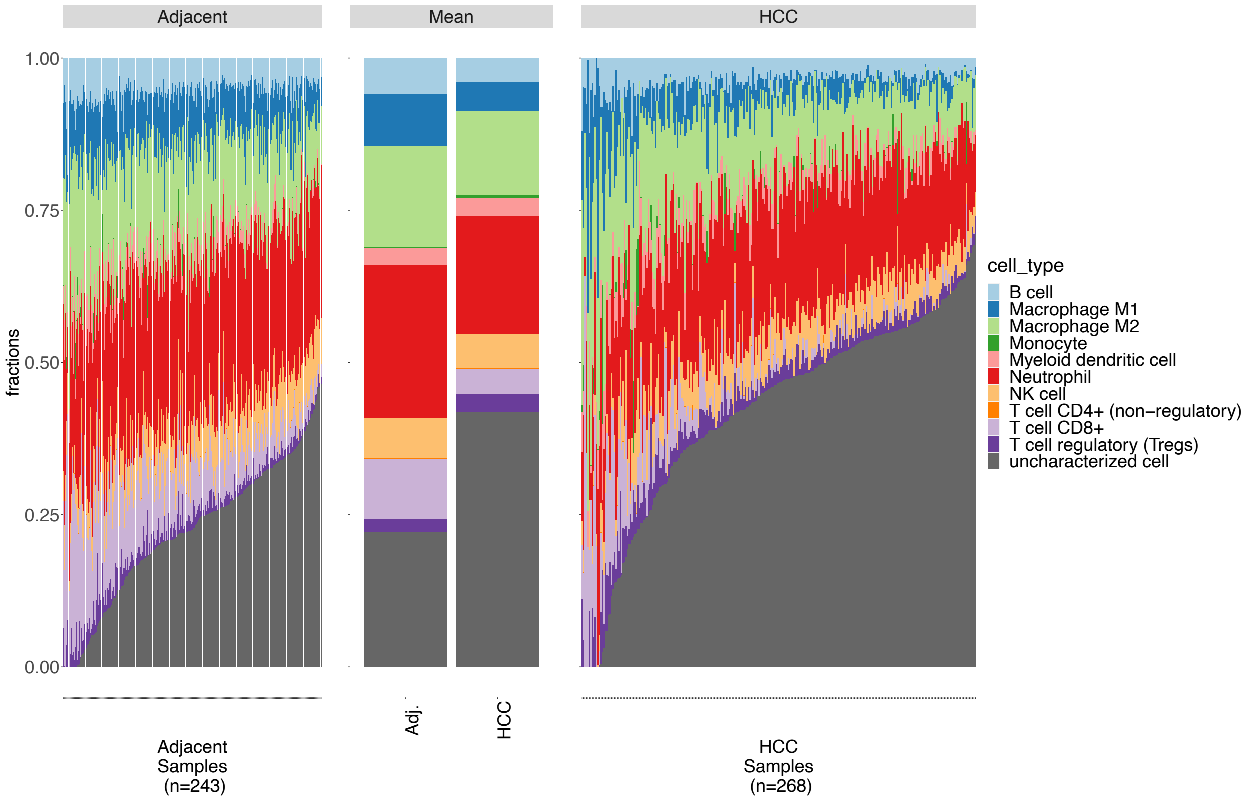

Figure S20: quanTIseq cell type fraction comparisons between adjacent and tumour groups in HCCDB3 dataset. Stacked bar charts of cell type fractions of adjacent and HCC groups are sorted in ascending order of uncharacterized cells (i.e., tumour and stromal) and aggregated by the mean proportion of cell types in each group.

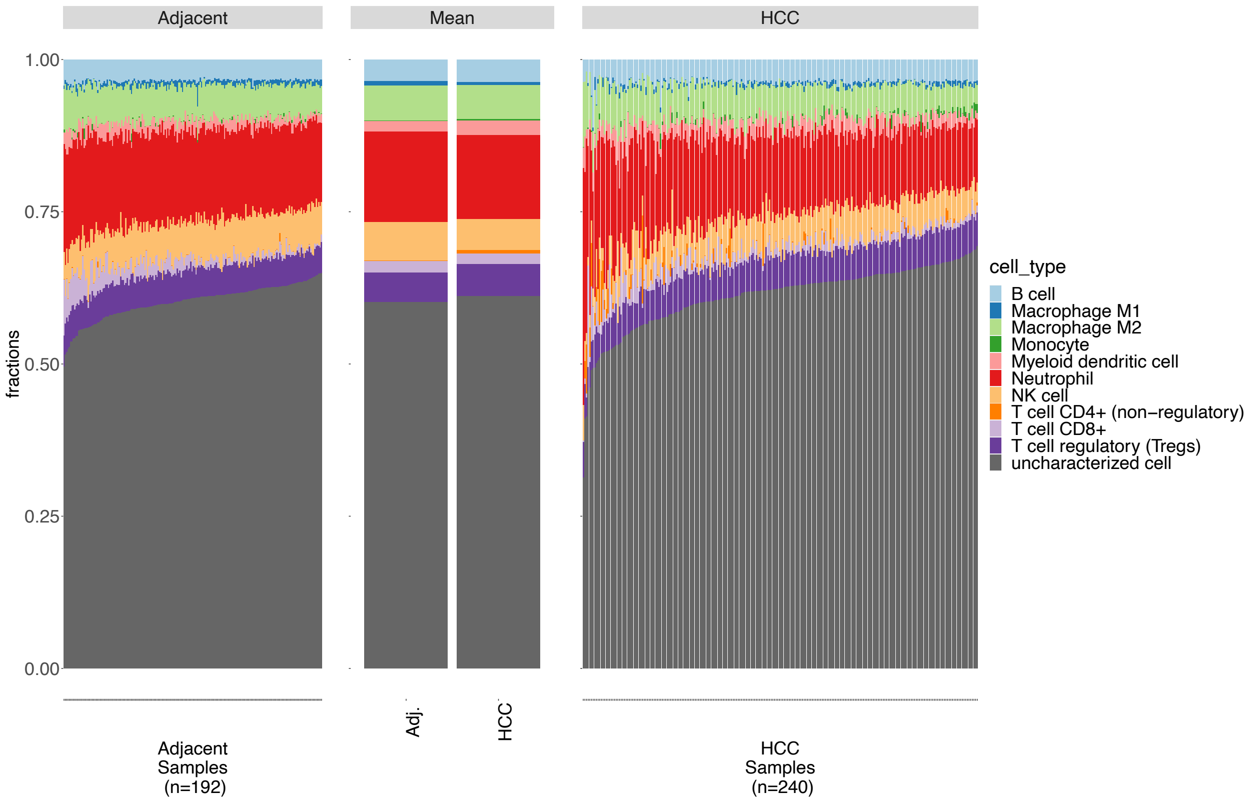

Figure S21: quanTIseq cell type fraction comparisons between adjacent and tumour groups in HCCDB4 dataset. Stacked bar charts of cell type fractions of adjacent and HCC groups are sorted in ascending order of uncharacterized cells (i.e., tumour and stromal) and aggregated by the mean proportion of cell types in each group.

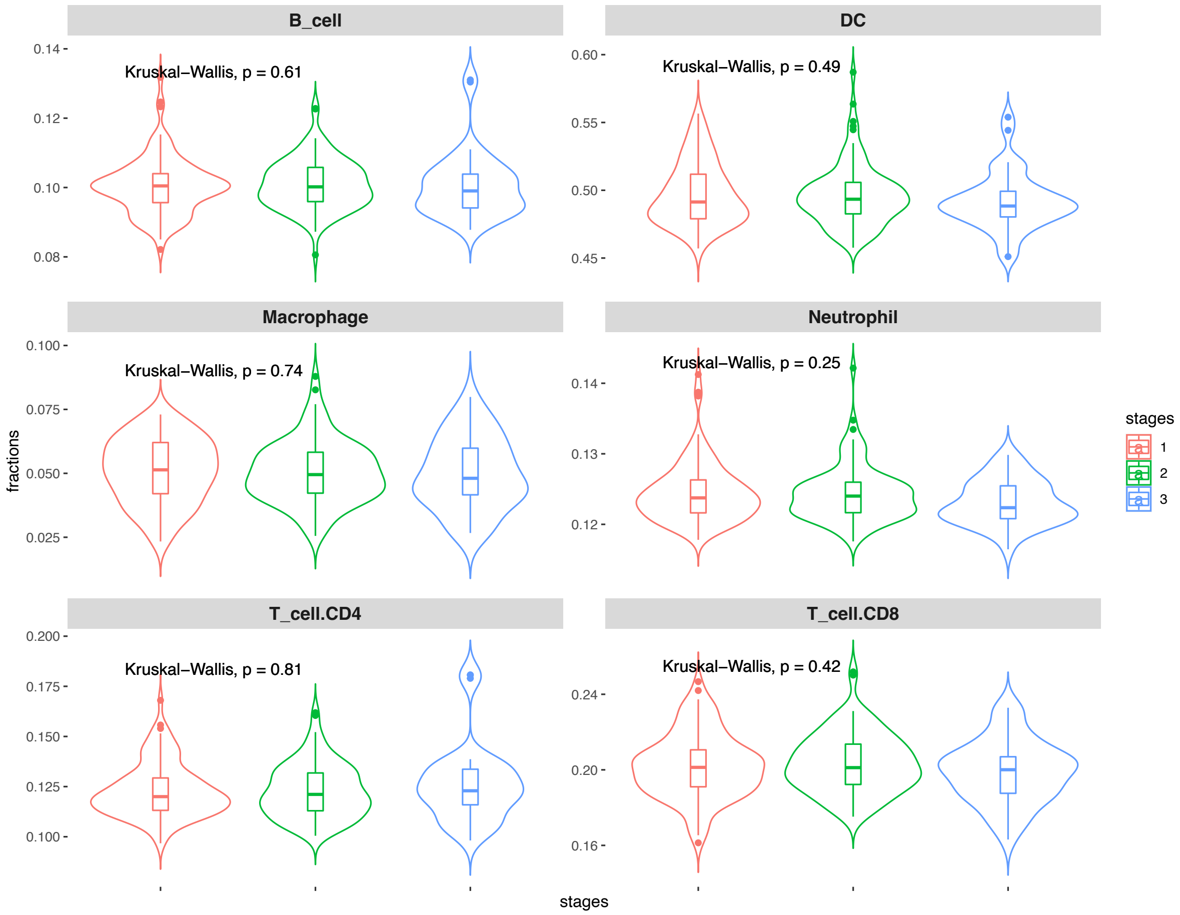

Figure S22: Kruskal-Wallis test for cell scores of tumour subtypes by TIMER. Violin box plots of TIMER show estimated abundance scores of cell types for tumour stages 1, 2, and 3 in HCCDB4 dataset. Kruskal Wallis tests were performed to compare three groups for each cell type with 0.05 significance level.
